# DNA Electrochemical Biosensors for In Situ Probing of Pharmaceutical Drug Oxidative DNA Damage

**DOI:** 10.3390/s21041125

**Published:** 2021-02-05

**Authors:** Ana-Maria Chiorcea-Paquim, Ana Maria Oliveira-Brett

**Affiliations:** 1 Department of Chemistry, Centre for Mechanical Engineering, Materials and Processes—CEMMPRE, University of Coimbra, 3004-535 Coimbra, Portugal; anachior@ipn.pt; 2 Instituto Pedro Nunes, Rua Pedro Nunes, 3030-199 Coimbra, Portugal

**Keywords:** DNA electrochemical biosensor, oxidative DNA damage, damage to DNA bases, biomarker of DNA damage, voltammetry, DNA oxidation, 8-oxoguanine (8-oxoG), 8-oxo-2′-deoxyguanosine (8-oxodG), 2,8-dihydroxyadenine (2,8-DHA)

## Abstract

Deoxyribonucleic acid (DNA) electrochemical biosensors are devices that incorporate immobilized DNA as a molecular recognition element on the electrode surface, and enable probing in situ the oxidative DNA damage. A wide range of DNA electrochemical biosensor analytical and biotechnological applications in pharmacology are foreseen, due to their ability to determine in situ and in real-time the DNA interaction mechanisms with pharmaceutical drugs, as well as with their degradation products, redox reaction products, and metabolites, and due to their capacity to achieve quantitative electroanalytical evaluation of the drugs, with high sensitivity, short time of analysis, and low cost. This review presents the design and applications of label-free DNA electrochemical biosensors that use DNA direct electrochemical oxidation to detect oxidative DNA damage. The DNA electrochemical biosensor development, from the viewpoint of electrochemical and atomic force microscopy (AFM) characterization, and the bottom-up immobilization of DNA nanostructures at the electrode surface, are described. Applications of DNA electrochemical biosensors that enable the label-free detection of DNA interactions with pharmaceutical compounds, such as acridine derivatives, alkaloids, alkylating agents, alkylphosphocholines, antibiotics, antimetabolites, kinase inhibitors, immunomodulatory agents, metal complexes, nucleoside analogs, and phenolic compounds, which can be used in drug analysis and drug discovery, and may lead to future screening systems, are reviewed.

## 1. Introduction

Deoxyribonucleic acid (DNA) is the most important element of the genetic apparatus, playing a dual role, carrying genetic information and regulating its expression. Moreover, DNA participates in complex interactions with different proteins and protein complexes, and is the principal target for many pharmaceutical drugs, including antibiotics and antineoplastic drugs.

**Scheme 1:**
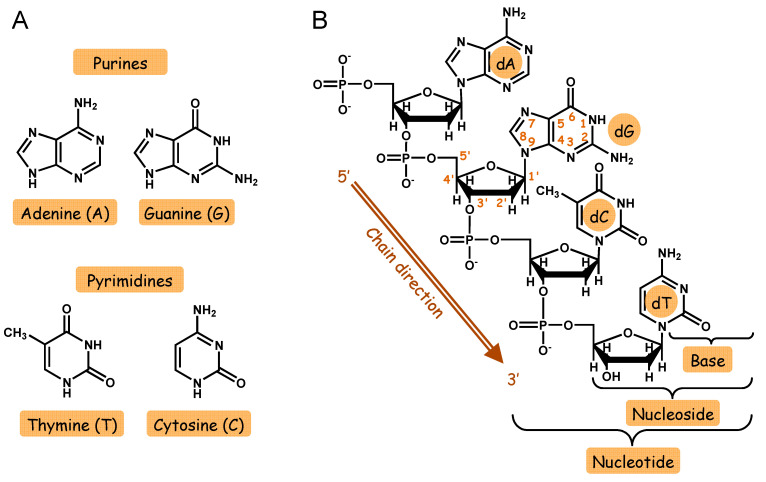
(**A**) Chemical structures of the deoxyribonucleic acid (DNA) aromatic bases, and (**B**) DNA fragment with the nucleosides sequence deoxyadenosine (dA), deoxyguanosine (dG), deoxythymidine (dT) and deoxycytidine (dC) linked by 3’,5’ -phosphodiester bonds. Chain direction is from 5′ to 3′ end. Atom numbering is presented in the nucleotide unit 5′-guanosine monophosphate (GMP).

The DNA basic repeating motif is the nucleotide, shown in Scheme 1, that is composed of a cyclic *β*-D-2′-deoxyribose sugar, phosphorylated at the 5′ position and substituted at Cl’, by one of four different aromatic bases attached by a *β*-glycosyl Cl’-N linkage. The four aromatic bases are adenine (A), guanine (G), thymine (T), and cytosine (C), as shown in Scheme 1. The G and A bases are more susceptible and more easily oxidized, as their oxidation potentials are less positive. Their oxidation in vivo can occur both by direct oxidation of G and A nucleotides and nucleosides in the genomic DNA, or by oxidation of deoxyguanosine monophosphate (dGMP) and deoxyadenosine monophosphate (dAMP) or guanosine monophosphate (GMP) and adenosine monophosphate (AMP) in the nucleotide pool.

The G base oxidation leads to the formation of a number of different oxidation products [1,2,3], the predominant forms being 8-oxoguanine (8-oxoG or 8-oxoGua), also known as 7,8-dihydro-8-oxoguanine, 8-dihydroguanine or 8-hydroxyguanine (8-OHG) [4]. Its corresponding nucleotide is 8-oxo-2′-deoxyguanosine (8-oxodG), also known as 8-oxo-7,8-dihydro-2′ -deoxyguanosine or 8-hydroxy-2′-deoxyguanosine(8-OHdG). A base oxidation also leads to the formation of a number of different oxidation products, the most important being 2,8-dihydroxyadenine (2,8-DHA). The G and A oxidation products can be further incorporated into DNA during replication or repair [5,6]. In particular, 8-oxoG and 2,8-DHA are highly mutagenic, and they are generally recognized as biomarkers of oxidative DNA damage and cellular oxidative stress.

**Scheme 2:**
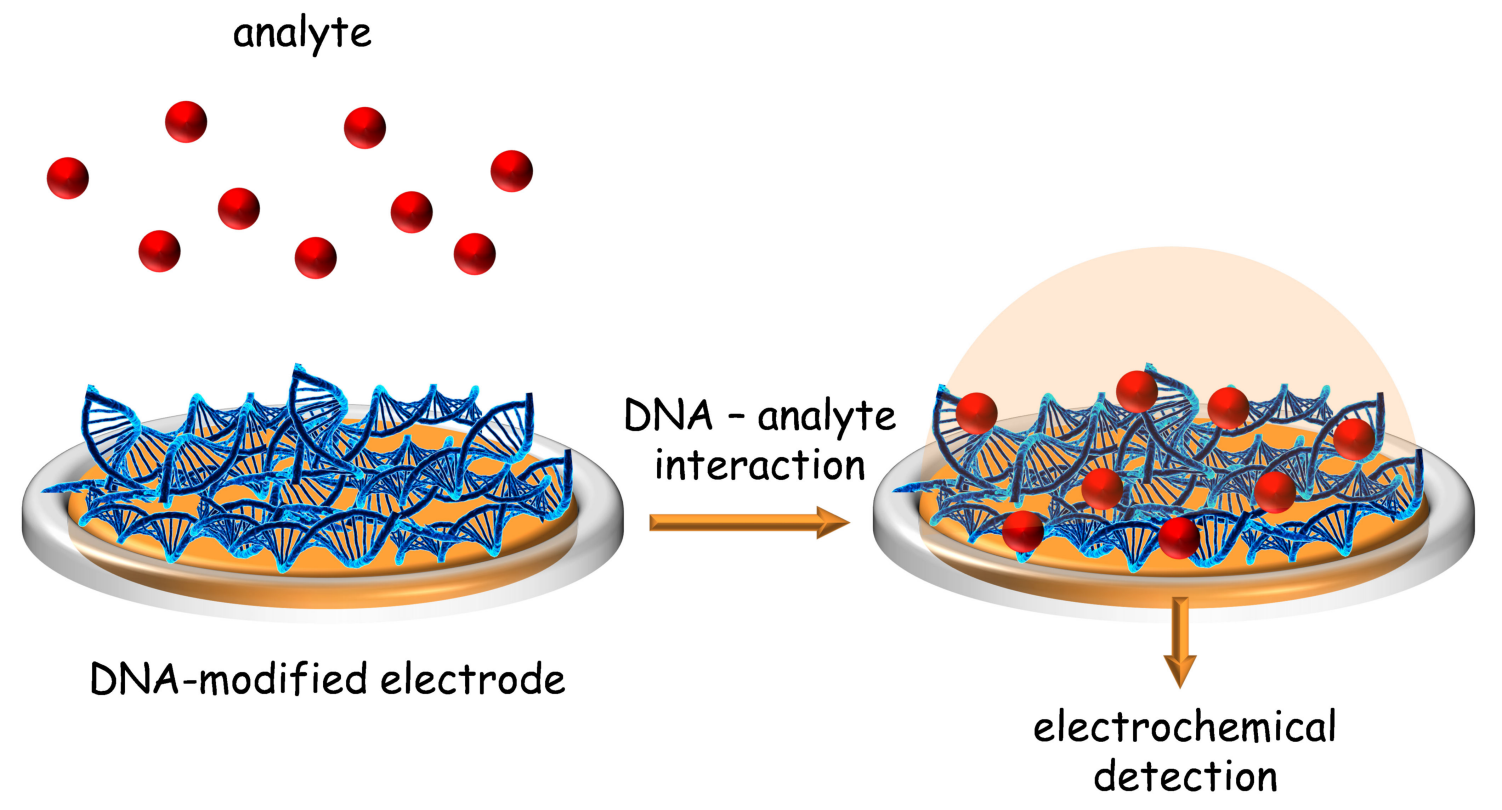
Schematic representation of DNA electrochemical biosensor design and operation. A DNA layer is immobilized at the electrode surface and acts as a recognition element, interacting with the target analyte; the DNA oxidation peaks before and after the interaction with the analyte are recorded and compared.

An electrochemical nucleic acid-based biosensor is defined by the International Union of Pure and Applied Chemistry (IUPAC) as a device that incorporates nucleic acids, i.e., natural and biomimetic forms of oligo- and polynucleotides, as a biological recognition element, and an electrode as a physicochemical transducer [7]. The applications of DNA electrochemical biosensors are based on the interaction of the analyte of interest with the DNA detection layer immobilized onto the electrode; the changes in the DNA structure, morphology, and electrochemical behavior, caused by the DNA–analyte interaction, are then detected at nanoscale [8,9,10,11,12,13,14,15,16,17,18,19], as shown in Scheme 2.

**Scheme 3:**
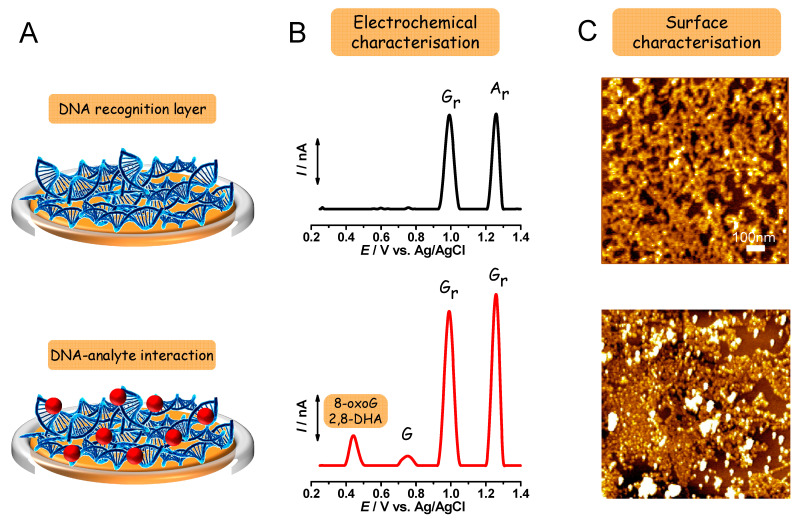
DNA electrochemical biosensor: (**A**) DNA recognition layer before and after interaction with the target analyte, and (**B**) electrochemical and (**C**) atomic force microscopy (AFM) characterization. The difference between voltammetric behavior and surface morphology of the control DNA and of the DNA–analyte complex is recorded and compared.

The label-free DNA electrochemical biosensor sensing is based on the direct detection of the oxidation peaks of the DNA constituents, nucleotides, nucleosides, and purine and pyrimidine bases, and of the oxidation peaks of 8-oxoG and 2,8-DHA biomarkers of the oxidative DNA damage. The difference between the anodic peaks of the intact control dsDNA and of the DNA–analyte complex is recorded and compared, as shown in Scheme 3B. The direct detection of the 8-oxoG and 2,8-DHA oxidation with the DNA electrochemical biosensor reveals the analyte’s ability to cause oxidative DNA damage. Moreover, the immobilized DNA, generally in a double-stranded configuration, suffers morphological changes after interacting with the target analyte (Scheme 3A), that can also be investigated, as shown in Scheme 3C.

The DNA electrochemical biosensor enables the study of the DNA interactions with pharmaceutical drugs [11,19] and proteins [20,21,22], and also foreseeing the damage caused to DNA integrity by hazardous carcinogenic compounds, such as metal ions [23,24,25] pollutants [19,26,27], free radicals [28,29,30], and ionizing radiation [31]. 

The discovery, development, and manufacture of pharmaceuticals require practical and low-cost analytical techniques for determination of the drugs in pharmaceutical formulations or in human biological fluids. Furthermore, a correct understanding of the drugs’ interaction mechanisms with the genomic DNA is required, in order to predict their structure–function mechanism of action. The DNA electrochemical biosensors present many biotechnological applications in pharmacology, due to their high sensitivity, short time of analysis, low cost, ability to achieve quantitative electroanalytical determination of drugs, and ability to determine in real time the DNA interaction mechanisms not only with the drugs, but also with their degradation products, their redox reaction products, and their metabolites in biological fluids. 

This review presents the design, development, and applications of label-free DNA electrochemical biosensors based on direct DNA electrochemical detection, to assess the oxidative DNA damage caused by pharmaceutical compounds, relevant for drug discovery and analysis. The electrochemical and atomic force microscopy (AFM) characterization of different bottom-up immobilization strategies of DNA nanostructured films onto electrodes are described, and the biosensors’ applications for a wide range of pharmaceutical compounds, ranging from antibiotics and anticancer drugs to antibodies, are reviewed. Although the applications of DNA electrochemical biosensors have been selectively reviewed, an exhaustive overview of the recent achievements in DNA electrochemical biosensors’ evaluation of pharmaceutical drugs, from the point view of their in situ probing of oxidative DNA damage, is still required. This review is of great interest to the chemistry, biochemistry, and medical communities, because the 8-oxoG and 2,8-DHA biomarkers of oxidative DNA damage and cellular oxidative stress represent important parameters in the context of the development of innovative actions and systems that enable disease prevention, early diagnosis, and treatment in healthcare. 

## 2. DNA Electrochemical Biosensor Characterization

### 2.1. Electrochemical Characterization

The development and correct application of DNA electrochemical biosensors requires a good characterization of the electrochemical response of the immobilized DNA recognition layer, and a correct assessment of the DNA morphology before and after the interaction with the analyte.

#### 2.1.1. Electrochemical Detection of DNA Base, Nucleoside, and Nucleotide Oxidation

**Figure 1:**
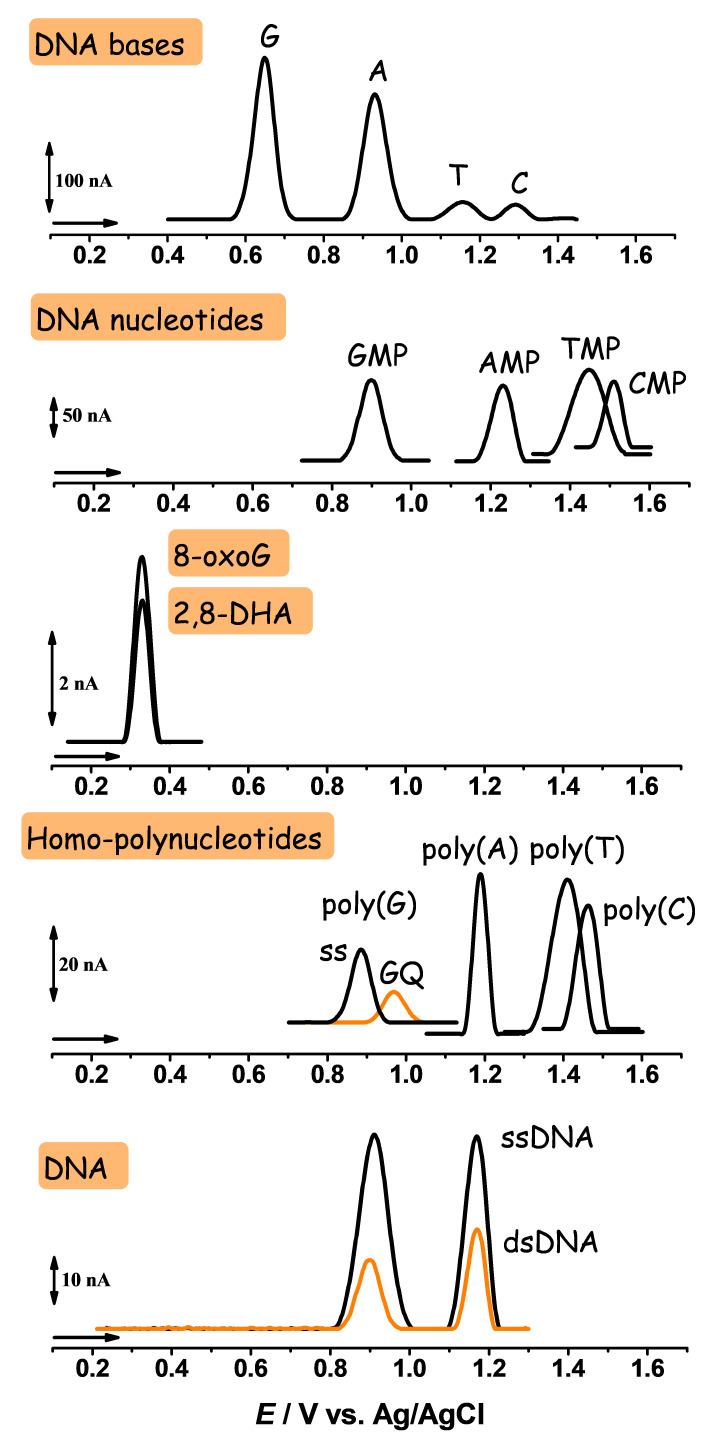
Baseline-corrected differential pulse voltammetry (DPV) with a glassy carbon electrode (GCE), in pH 7.0 solutions of: 20 µM equimolar mixture of G, A, T, and C, 20 µM GMP, 20 µM AMP, 500 µM TMP, 500 µM CMP, 0.5 μM 8-oxoG, 0.5 μM 2,8-DHA, 5 μg mL^−1^ poly(G), 40 μg mL^−1^ poly(A), 100 μg mL^−1^ poly(T), 100 μg mL^−1^ poly(C), 60 μg mL^−1^ dsDNA, and 60 μg mL^−1^ ssDNA. Adapted from [11,18,32,33,34,35] with permission.

Oxidation in the genomic DNA can result in damage to the DNA bases, nucleosides, and nucleotides. The G base presents the lowest oxidation potential at carbon electrodes, at *E*_p_ ~ +0.70 V, followed by A at *E*_p_ ~ +0.96 V, T at *E*_p_ ~ +1.16 V, and C at *E*_p_ ~ +1.31 V (vs. Ag/AgCl, 3 M KCl), at pH = 7.4 [32], as shown in Figure 1.

Differential pulse voltammetry (DPV) showed that G oxidation at a glassy carbon electrode (GCE) is a two-step process: the first step is correlated with the irreversible oxidation of G to 8-oxoG that takes place with the transfer of two electrons and two protons, and the second step is correlated with the irreversible oxidation of G dimers that takes place with the transfer of one electron [36]. A oxidation is irreversible and takes place in two steps: the first is correlated with the A oxidation and the second one with the oxidation of A dimers [36].

The oxidation of DNA nucleosides, deoxyadenosine (dA), deoxyguanosine (dG), deoxythymidine (dT) and deoxycytidine (dC), occurs at the same potentials as the nucleotides, GMP, AMP, thymidine monophosphate (TMP) and cytidine monophosphate (CMP), as shown in Figure 1, with dG and GMP at *E*_p_ ~ +0.95 V, dA and AMP at *E*_p_ ~ +1.21 V, dT and TMP at *E*_p_ ~ +1.41 V, and dC and CMP at *E*_p_ ~ +1.56 V (vs. Ag/AgCl, 3 M KCl), at pH = 7.4, being shifted to more positive potentials, compared with the bases’ oxidation potentials [32]. As the sugar and phosphate sugar groups are not oxidized in aqueous solutions, the shift is associated with a more difficult electron transfer from the bases inside the nucleotides and nucleotides’ bulkier structures to the electrode.

#### 2.1.2. Electrochemical Detection of 8-oxodG and 2,8-DHA Biomarkers Oxidation 

**Scheme 4:**
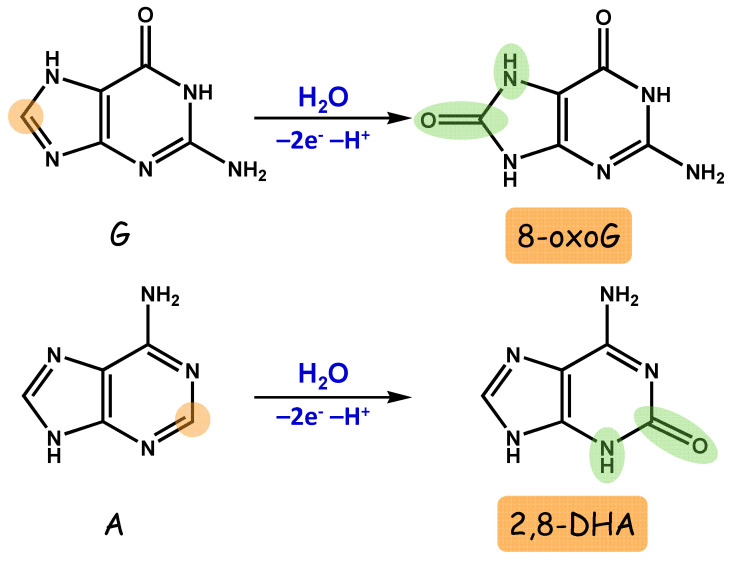
Oxidation of G to 8-oxoG, and of A to 2,8-DHA.

The 8-oxoG is the G base oxidation product at the C8 position, and presents several tautomeric forms in aqueous solution, the most stable being the 6,8-diketo I tautomer, with an oxo group at the C8 position and a H atom at the N7 positions, as shown in Scheme 4 [37,38,39,40]. The electrochemical oxidation mechanisms of G and guanosine at carbon electrodes, with the formation of 8-oxoG and 8-oxodG, have been extensively studied under different experimental conditions [2,4,10,11,13,16,17,18,29,32,36,41,42,43,44,45].

The 8-oxoG redox behavior at a GCE was studied by cyclic voltammetry (CV), DPV, and square wave voltammetry (SWV), showing a pH-dependent oxidation mechanism that takes place with the transfer of two electrons and two protons [35]. Moreover, 8-oxoG is more easily oxidized than G, presenting a lower oxidation potential, at *E*_p_ ~ +0.30 V, when compared with G, at *E*_p_ ~ +0.70 V (vs. Ag/AgCl, 3 M KCl), for the same experimental conditions, at pH = 7.4 [35,46], as shown in Figure 1, thus making it possible to electrochemically distinguish 8-oxoG from G in real samples.

The 8-oxodG oxidation mechanism is comparable among different electrode materials [2,29,47,48], involving a two-electron two-proton charge transfer step, followed by irreversible chemical reactions, with the rate of the charge transfer reaction step depending on the electrode material and following the sequence GCE > Pt, Au >> SnO_2_.

Normally, the 8-oxoG determinations in urine real samples cause a number of difficulties, due to the presence of uric acid (UA), a purine structurally very similar to 8-oxoG, which is electroactive and is excreted in urine at concentrations of ~ 0.5 mM (25–70 mg L^−1^), 10^4^ times higher than 8-oxoG and 8-oxodG urine levels. The 8-oxoG electrochemical oxidation in the presence of UA interferents has been investigated by DPV in a pH range of 1 to 12 [46]. The difference between the oxidation peak potentials of 8-oxodG and UA is less than 100 mV in the whole pH range, and the separation is greater for the pH interval 4–7. In solutions of 8-oxodG and UA mixtures, pH = 6 represents the optimum pH for 8-oxoG determination in the presence of UA, since the peak current is higher and a larger peak separation is achieved [46].

#### 2.1.3. Electrochemical Detection of DNA Oxidation

Natural cellular DNA generally adopts a double-stranded DNA (dsDNA) configuration, with Watson–Crick base pairs. However, a variety of other conformations and structural motifs can also be present, such as single-stranded DNA (ssDNA), triplex helixes, four-stranded secondary structures (e.g., *i*-motifs formed by C-rich DNA sequences, or G-quadruplexes (GQs) formed by G-rich DNA sequences found in the chromosomes’ telomeric regions), stem-loops (hairpin loops), cruciform DNA, D-loops, etc. [18,49,50]. The electrochemical methods are able to distinguish between different DNA secondary structures, and have been employed to study the DNA structural modifications, such as transitions from/to single- and double-stranded and GQ secondary structures.

DPV of dsDNA at a GCE shows a pH-dependent behavior, with two small oxidation peaks, associated with G residue (G_r_) and A residue (A_r_) oxidation in the dsDNA structure, as shown in Figure 1 [8,12,16,18,33]. T residue (T_r_) and C residue (C_r_) oxidation in the dsDNA structure presents low anodic currents and occurs at very high positive potentials, near the oxygen evolution, and is therefore not usually detected. 

DPV of ssDNA at a GCE also shows the G_r_ and A_r_ oxidation peaks (Figure 1) but with much higher currents compared with dsDNA. The ssDNA helix is more loose and less compact than dsDNA, and the G and A base residues reach the electrode more easily, thus enabling the electron transfer [14].

Long-chain homo-polynucleotides containing only one type of base are common in the DNA and RNA of human and animal genomes, and are generally employed in DNA electrochemical biosensors used for screening the analyte interaction with a specific DNA base. The poly(G), poly(A), poly(T), and poly(C) homo-polynucleotides present single-stranded configurations, with their oxidation being correlated with the oxidation of G_r_, A_r_, T_r_, and C_r_, respectively, as shown in Figure 1. 

The poly(G) can also form four-stranded GQ secondary structures, that consist of superposed G-quartets (planar association of four G bases interacting by Hoogsteen hydrogen bonds), stacked by π–π hydrophobic interactions and stabilized by monovalent cations such as K^+^ and Na^+^ placed in between the GQ planes [51,52]. In fresh solutions, poly(G) is mainly in a single-stranded configuration, and DPVs at a GCE show only the G_r_ oxidation peak [34], as shown in Figure 1. For longer incubation times, the poly(G) single strands self-assemble into QGs, with this process being correlated with the decrease and disappearance of the G_r_ oxidation peak and the appearance of a new GQ oxidation peak at a higher potential. The GQ oxidation peak is incubation time-dependent, presenting a maximum after 10 days and steady values after ~17 days [34], as shown in Figure 1. The poly(G) structural modifications from single-stranded into GQ configurations was confirmed by AFM.

**Figure 2:**
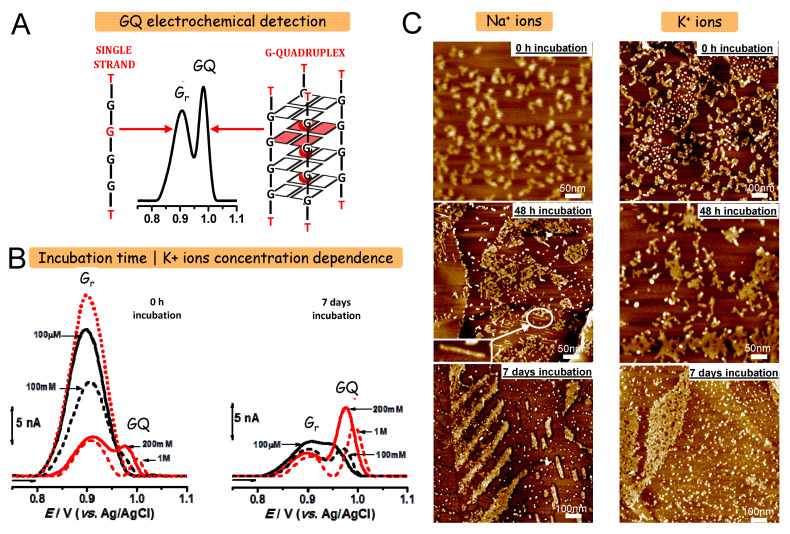
(**A**) Schematic representation of the d(TG_4_T) single-stranded and G-quadruplex (GQ) electrochemical detection. (**B**) Baseline-corrected DPV in 3.0 μM d(TG_4_T) at pH = 7.0, after 0 h and 7 days of incubation, in the absence of K^+^ ions (red dotted line), and in the presence of different concentrations of K+ ions. (**C**) AFM images of d(TG_4_T) spontaneously adsorbed onto highly oriented pyrolytic graphite (HOPG) from 0.3 μM d(TG_4_T) at pH = 7.0, after different incubation times. Adapted from [55] with permission.

The transition from single-stranded into GQ secondary structures was also observed by DPV at a GCE for a number of short-chain oligodesoxyribonucleotides (ODNs): d(G)_10_, d(TG_9_), d(TG_8_T) [22,52,53], the telomeric d(TG_4_T) repeat sequence, and the thrombin-binding aptamers (TBAs) d(G_2_T_2_G_2_TGTG_2_T_2_G_2_) and d(G_3_T_2_G_3_TGT_3_T_2_G_3_) [54], as shown in Figure 2A [55]. 

### 2.2. Morphological Characterization

The key parameters for the development of DNA electrochemical biosensors are the electrode material and the surface coverage, which directly influence the sensor response. A DNA electrochemical biosensor built with a thick multilayer DNA film has the electrode surface completely covered by DNA, thus preventing undesired analyte nonspecific adsorption onto the electrode [56], with the biosensor response corresponding exclusively to the DNA–analyte complex oxidation peak, without interference from the free analyte oxidation peak [11,56].

To determine the DNA adsorption onto carbon electrodes and the DNA recognition layer morphological characteristics, voltammetric and electrochemical impedance spectroscopy (EIS) methods were employed together with other analytical techniques, such as ellipsometry, surface enhanced Raman spectroscopy, and, more recently, AFM [10,12,17,57,58,59,60,61,62,63,64].

**Figure 3:**
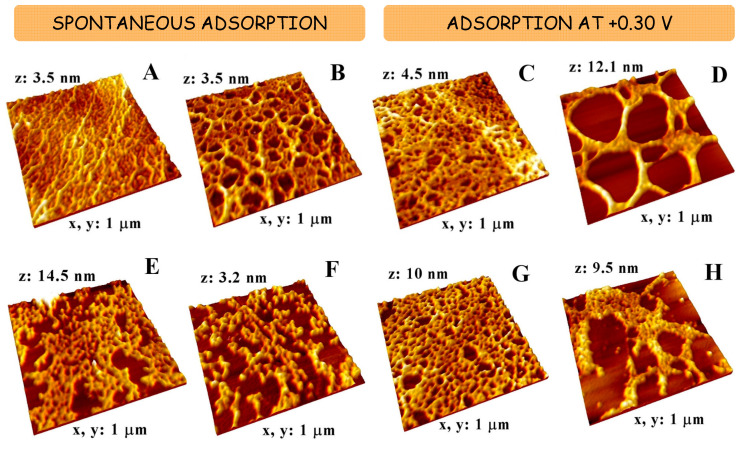
AFM images of the HOPG electrode modified by: **I.** (**A**–**D**) 60 μg mL^−1^ dsDNA (**A**,**B**) adsorbed spontaneously and (**C**,**D**) at applied potential of +0.30 V (vs. AgQRE), and **II.** (**E**–**F**) 5 μg mL^−1^ ssDNA adsorbed (**E**,**F**) spontaneously and (**G**,**H**) at applied potential of +0.30 V (vs. AgQRE), (**A**,**C**,**E**,**G**) pH 5.3 and (**B**,**D**,**F**,**H**) pH 7.0. Adapted from [58] with permission.

The AFM surface morphological characteristics of short- and long-chain DNA sequences adsorbed spontaneously and under electrochemical applied potential onto carbon electrodes were determined [57,58,63]. Long-chain dsDNA and ssDNA spontaneously adsorb onto highly oriented pyrolytic graphite (HOPG) as thin and uneven network films, with their morphology being dependent on the DNA concentration and solution pH, as shown in Figure 3A,B,E,F. A positive applied potential of ~+0.30 V (vs. silver wire as a quasi-reference electrode (AgQRE)), which increases the electrostatic interaction between the DNA phosphate backbone and the carbon electrode without oxidizing the G and A residues, leads to the formation of more robust and stable dsDNA and ssDNA films, as shown in Figure 3C,D,G,H.

The poly(G) adsorption morphology and changes in the secondary structure were investigated in Na^+^ or K^+^ cations containing solutions, using AFM at HOPG and DPV at a GCE [34]. After short incubation times, the poly(G) single strands self-assemble into small GQ regions that do not significantly influence the adsorption process. However, after long incubation times, bulky poly(G)-GQ aggregates are formed that interact and adsorb less onto the hydrophobic HOPG, since the G bases within the GQs are more protected and less capable of undergoing hydrophobic interactions.

The short-chain ODN structural variations influence their adsorption onto carbon electrodes, and therefore the DNA biosensor electrochemical response. Under physiological pH, the homo-ODNs d(A)_10_, d(C)_10_, and d(T)_10_ are single-stranded and spontaneously absorb onto HOPG as networks with a knotted and twisted appearance [50]. In mild acid pH solutions, the d(A)_10_ sequences are in double-stranded configurations and adsorb onto carbon electrodes as network films; moreover, the AFM showed that d(A)_10_ double strands adsorb less than the d(A)_10_ single strands, confirmed by DPV by the decrease in the A_r_ oxidation peak current. The d(C)_10_ can form *i-motifs* that were detected by AFM as globular aggregates [50].

The adsorption of d(G)_10_, d(TG_9_), d(TG_8_T) [22,52,53], and d(TG_4_T) [55], which can self-assemble into tetra-molecular GQ structures, was correlated with their redox behavior at a GCE. AFM and DPV showed that the GQ formation is dependent on the ODN sequence and concentration, pH, and Na^+^ and K^+^ monovalent cations. In the presence of Na^+^ cations for short incubation times, the ODN single strands absorb as networks and polymeric structures, and only the G_r_ oxidation peak was observed, as shown in Figure 2. In the presence of Na^+^ for long incubation times, or in the presence of K^+^ cations for all incubation times, the ODN single strands started to self-assemble into GQs, a time-dependent process observed with AFM by the presence of spherical aggregates and with DPV by the G_r_ oxidation peak decrease and the GQ oxidation peak’s appearance, increase, and shift to more positive potentials, as shown in Figure 2 [22,50]. 

The bottom-up immobilization of DNA with different structural configurations, such double- and single-strands, GQs, *i-motifs*, or large nanostructures, allows various applications. Short-chain ODNs and long-chain dsDNA, ssDNA, poly(G), and poly(A), in single-stranded, double-stranded, and GQ configurations, were successfully employed in DNA electrochemical biosensor applications for in situ probing the oxidative DNA damage caused by pharmaceutical drugs. 

## 3. DNA Electrochemical Biosensors for the Detection of Pharmaceutical Drugs

The DNA electrochemical biosensors’ high sensitivity has attracted considerable attention in pharmaceutical analytical applications [65], with them being used in the analysis of drugs, medicines, and other pharmaceutical compounds, and showing promising results in drug analysis and drug discovery.

### 3.1. Acridine Derivatives

Acridine derivatives are important compounds in medicinal chemistry due to their anticancer and antimicrobial properties. The polyaromatic ring of acridines has a high affinity for dsDNA, leading to intercalation and stacking. 

The trisubstituted acridine compound BRACO-19 was synthetized as a pharmaceutical anticancer agent, its mechanism of action being based on blocking the telomeric DNA into a GQ structure, thus inhibiting the telomerase. More recently, GL15 and GL7 triazole-linked acridine ligands, with improved binding selectivity for human telomeric GQ DNA over dsDNA, have been designed, synthetized, and evaluated. The BRACO-19, GL15, and GL7 oxidation mechanisms at a GCE are pH dependent, irreversible, and adsorption controlled [66,67]. DPV studies in incubated solutions and at dsDNA, poly(G), and poly(A) electrochemical biosensors [67] showed that GL15 and GL7 interact specifically with the G-rich segments of the DNA, causing time-dependent DNA condensation, without oxidative DNA damage. 

**Figure 4:**
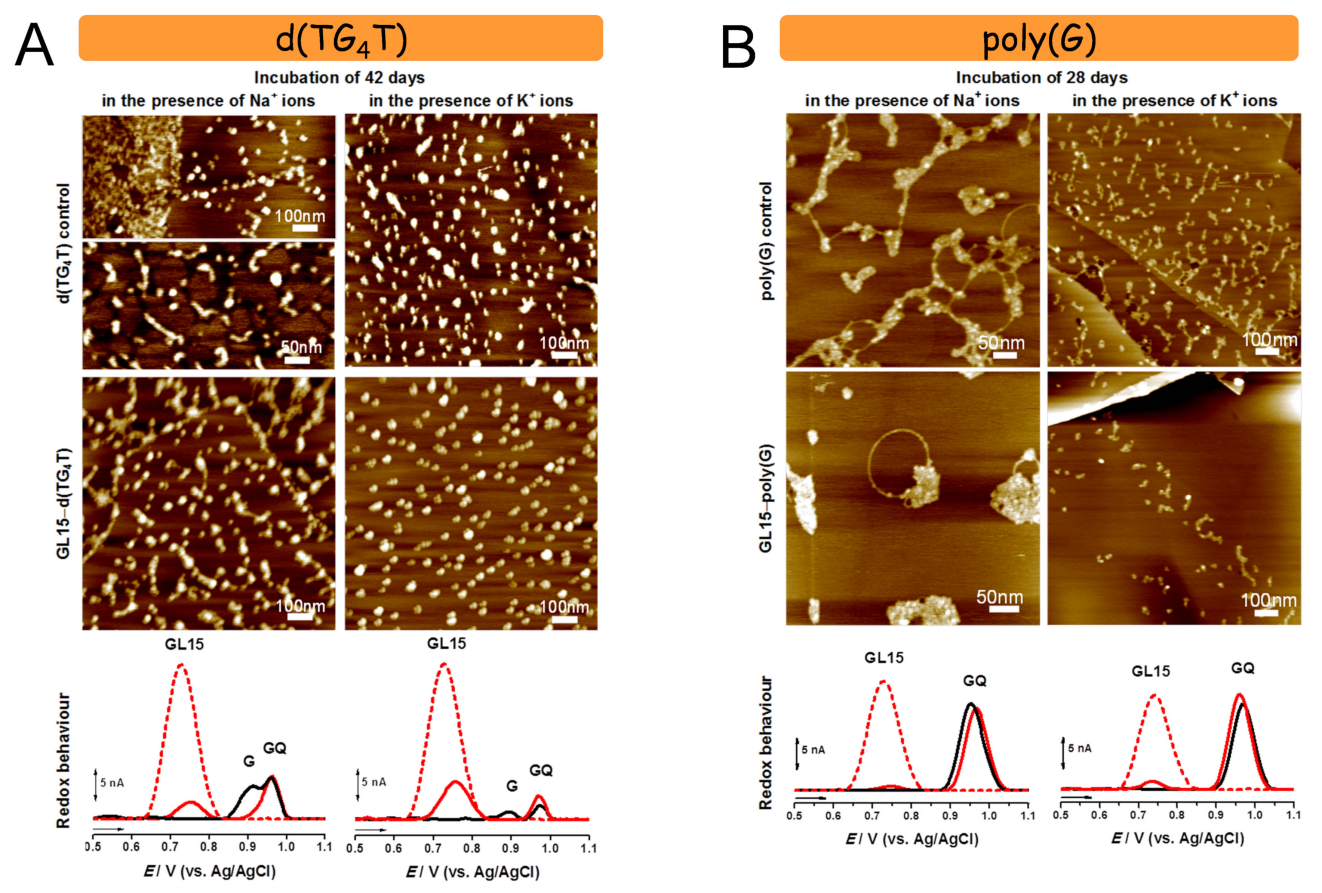
AFM images and baseline-corrected DPV of (**A**) d(TG4T) control and GL15-d(TG4T) and (**B**) poly(G) control and GL15-poly(G), in Na^+^ and K^+^ ion-containing solutions: (black line) DNA control, (red dotted line) GL15 control, and (red line) GL15-DNA. Adapted from [68] with permission.

The GL15 interactions with the telomeric DNA sequence d(TG_4_T) from *Tetrahymena* and with poly(G) have been investigated [68]. GL15 induces GQ formation and stabilization: AFM showed the adsorption of aggregates, which were small and globular in the case of d(TG_4_T), and large in the case of poly(G), whereas DPV showed the decrease and disappearance of the GL15 and G_r_ oxidation peaks and the appearance of the GQ oxidation peak, as shown in Figure 4. In both Na^+^ and K^+^ ion-containing solutions, the GL15 interaction with d(TG_4_T) and poly(G) is time dependent, takes place at the terminal G-quartets of the GQs, stabilizing and accelerating their formation. In the presence of K^+^ cations, d(TG_4_T) self-assembles into compact and fully aligned tetra-molecular GQs, while in the presence of Na^+^ cations, the growth of small nanowires, induced by the presence of non-aligned QGs, was observed [68].

Amsacrine is an aminoacridine derivative with potential antineoplastic activity, due to its intercalation into DNA that further inhibits topoisomerase II, resulting in dsDNA breaks, arrest of the S/G2 phase of the cell cycle, and cell death. An electrochemical biosensor, based on the dsDNA immobilization onto an Eu^3+^-doped NiO carbon paste electrode (CPE), for screening the amsacrine, was developed [69]. The dsDNA-amsacrine interaction was investigated by DPV, UV–Vis spectrophotometry, and docking techniques. Based on the G_r_ oxidation peak current, amsacrine electrochemical detection in the linear range 0.1–100.0 mM, with a limit of detection (LOD) of 0.05 mM and with reasonable selectivity in the presence of interferences, was achieved. The sensor was also successfully tested in human serum and urine samples.

The DNA interaction with a hybrid anticancer drug 7ESTAC01, composed of acridine and thiophene anticancer pharmacophores, was investigated [70]. The biosensor consisted of a stem-loop DNA probe covalently attached to a gold electrode that hybridized with a complementary DNA sequence, to form a dsDNA sequence. Following the 7ESTAC01-DNA interaction, changes in the G_r_ and A_r_ oxidation peak currents were observed, and the 8-oxoG oxidation peak appeared for high 7ESTAC01 concentrations, correlated with the oxidative DNA damage. The intercalation of the aminothiophene and partial acridine rings in between the DNA bases was demonstrated using molecular docking, density-functional theory studies, and UV–Vis spectroscopy. 

### 3.2. Alkaloids

Topotecan is a semisynthetic derivative of camptothecin, a cytotoxic, quinoline-based alkaloid extracted from the Asian tree *Camptotheca acuminate*. Topotecan is used as an antineoplastic agent in the therapy of cervical, colorectal, ovarian, and small cell lung cancer, acting through the inhibition of topoisomerase I activity. Topotecan electrochemical detection with a DNA electrochemical biosensor, based on dsDNA immobilization onto a CPE modified by ionic liquid and graphene quantum dots, was investigated [71]. Topotecan detection by DPV, in the linear range 0.35–100.0 μM, with an LOD = 0.1 μM, was obtained. The system has also been tested for topotecan detection in blood serum and urine samples.

A ds-DNA/Eu^3+^-doped NiO/CPE electrochemical biosensor for screening the anticancer drug topotecan was also made, showing a linear range of 0.09–110.0 μM, and LOD = 0.04 μM [72].

Similar to topotecan, irinotecan is a semisynthetic derivative of the *Camptotheca acuminate* tree, being used as antineoplastic agent in the therapy of colorectal, ovarian, and non-small cell lung cancer. A DNA electrochemical biosensor based on an electrodeposited cetyl trimethylammonium bromide-multiwalled carbon nanotube (CN) composite on single-use graphite electrodes was used to evaluate the irinotecan–DNA interaction [73]. Based on the G_r_ oxidation peak current, by DPV, irinotecan detection was achieved in the linear range 2–500 μg mL^−1^ with LOD = 1.03 μg mL^−1^, and an equilibrium constant of K = 6.84 × 104 M^−1^ for the binding process was determined. The sensor was also applied for irinotecan quantitative determination in serum samples. 

Etoposide is a semisynthetic derivative of podophyllotoxin extracted from the mandrake root *Podophyllum peltatum*, which is used as antineoplastic agent in the therapy of several forms of solid tumors, leukemia, and lymphoma. An electrochemical DNA biosensor for etoposide detection, based on the electrostatic adsorption of DNA onto a ternary nanocomposite containing layered double hydroxides, cobalt ferrite, and graphene oxide, electrophoretically deposited on a fluorine tin oxide substrate, was developed [74]. Etoposide electroanalytical determination, in the linear range 0.2–10 µM, with LOD = 0.0010 µM, was achieved, and the biosensor maintained reproducibility and stability of about 95% of the initial activity after 6–7 weeks, and showed good results in human blood plasma, serum, and urine, with good 97.0–104.0% recoveries.

### 3.3. Alkylating Agents

Temozolomide, commonly known under the brand name Temodal, is an antineoplastic alkylating agent generally used against aggressive types of brain tumors. CV and DPV studies showed that temozolomide reduction is a pH-dependent irreversible process that occurs at the tetrazinone ring, causing its irreversible breakdown, while temozolomide oxidation is a pH-dependent, two-step irreversible process [75,76]. The dsDNA interaction with temozolomide and with its metabolites, 5-aminoimidazole-4- carboxamide (AIC) and methyldiazonium ion, has also been studied [77], showing a time-dependent decrease in the G_r_ and A_r_ oxidation peaks, consistent with dsDNA condensation. The temozolomide and AIC/methyldiazonium ion interactions with dsDNA at a multilayer dsDNA electrochemical biosensor confirmed the dsDNA condensation and demonstrated that the interaction involved mostly the G residues. The temozolomide metabolites induced oxidative DNA damage, detected by monitoring the existence of 8-oxoG/2,8-DHA biomarker oxidation peaks. 

A DNA electrochemical biosensor for the detection of temozolomide, based on a pencil graphite electrode (PGE) modified with Au nanoparticles and dsDNA, was also developed [78]. Temozolomide detection in the linear range 5.0 nM–45.0 μM, with LOD = 1.0 nM, was observed. A docking investigation was further used to evaluate the temozolomide intercalation between the G bases in the minor grooves. Theoretical studies confirmed that the temozolomide intercalated into the dsDNA is stabilized by π–π interactions.

Lomustine is a bifunctional alkylating antineoplastic agent that belongs to the nitrosurea class, and is used to treat brain tumors. The dsDNA in situ interaction with lomustine and with chemically degraded lomustine was investigated in incubated solutions, using dsDNA electrochemical biosensors and comet assays [79]. Lomustine metabolites initially cause condensation of the DNA double helix, followed by DNA unwinding. Free G bases are released from the dsDNA, and oxidative damage to the DNA by the lomustine metabolites was seen, via the detection of 8-oxoG and 2,8-DHA oxidation peaks. Using poly(dA) and poly(dG) electrochemical biosensors, it was demonstrated that the lomustine metabolites caused oxidative DNA damage to both G and A residues.

### 3.4. Alkylphosphocholines

**Figure 5:**
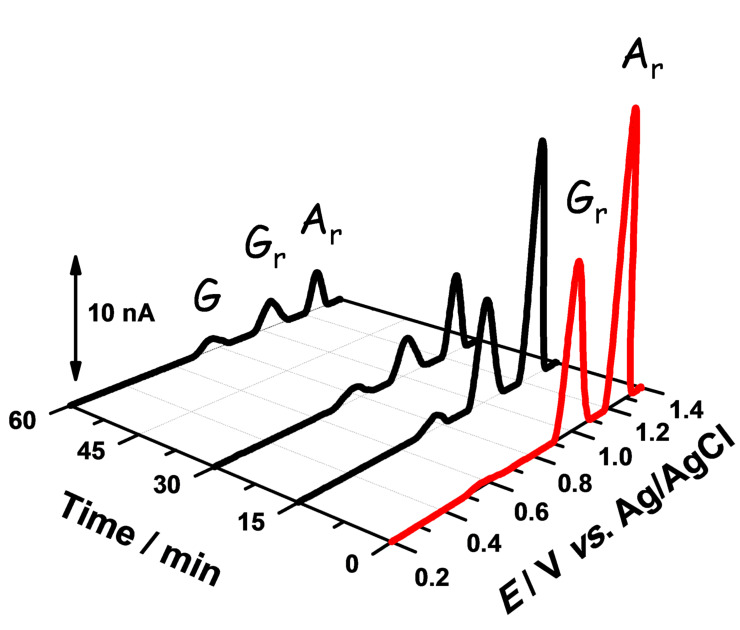
Baseline-corrected DPV at pH 4.5, obtained with a dsDNA–electrochemical biosensor, (red line) before and (black line) after incubation with 0.1 μM miltefosine, during different time periods. Adapted from [80] with permission.

Miltefosine belongs to the class of alkylphosphocholine drugs, and presents activity against various parasite species, pathogenic bacteria and fungi, and cancer cells, and is currently used to treat visceral, cutaneous, and mucosal forms of the parasitic disease leishmaniasis. The miltefosine–dsDNA interaction was investigated with a GCE, in incubated solutions and at dsDNA electrochemical biosensors [80], by following the changes in the G_r_ and A_r_ oxidation peaks, and the appearance of the free G oxidation peak, as shown in Figure 5. The DPV results showed that the miltefosine–dsDNA interaction occurs either independently of the dsDNA sequence, leading to the condensation/aggregation of DNA strands and producing a rigid miltefosine–dsDNA complex, or preferentially at the G bases, causing the release of free G. However, miltefosine did not cause oxidative DNA damage.

### 3.5. Antibiotics

Anthracycline antibiotics are an important class of antitumor drugs, initially synthesized from *Streptomyces bacterium*; they are widely used in cancer chemotherapy to treat solid tumors and hematological malignancies [81].

Doxorubicin, sold under the brand name Adriamycin, among others, is a chemotherapy drug used to treat breast cancer, bladder cancer, Kaposi’s sarcoma, lymphoma, and acute lymphocytic leukemia. Doxorubicin adsorption and electrochemical behavior were studied by AFM and DPV [82,83,84], showing that doxorubicin’s 5,12-diquinone group is irreversibly reduced at a GCE, at *E*_p_ ~ −0.4 V and *E*_p_ ~ −0.6 V, while the 6,11-dihidroquinone-functionality is reversibly oxidized, at *E*_p_ ~ +0.5 V (vs. Ag/AgCl, 3 M KCl) [82]. Studies concerning the doxorubicin–DNA in situ interaction with a thick-layer dsDNA electrochemical biosensor, after applying a negative potential of −0.60 V for 60 s, confirmed the occurrence of oxidative DNA damage, detected via the appearance of the 8-oxoG oxidation peak. The applied negative potential leads to the reduction of the doxorubicin intercalated into the dsDNA, and to the formation of a doxorubicin radical that is responsible for the DNA oxidative damage. A mechanism for the oxidative DNA damage caused by doxorubicin was proposed [82].

A DNA biosensor for the detection of doxorubicin, based on the GCE modification with DNA immobilized between two layers of electropolymerized polyaniline, was also developed [85]. The DNA sensor was first incubated in a methylene blue solution, which amplified the signal due to DNA intercalation and competition with the doxorubicin molecules for the DNA binding sites. Using this procedure, doxorubicin quantification in the linear range 1.0 pM–0.1 µM, with LOD = 0.6 pM, was achieved. The DNA sensor was tested for the analysis of spiked artificial urine samples and showed satisfactory recovery in the concentration range 0.05–10 µM. 

Idarubicin is a semisynthetic analog of the antineoplastic anthracycline antibiotic daunorubicin, and is used for different types of leukemia. Idarubicin is irreversibly oxidized at a GCE, in a pH-dependent, one-electron and one-proton transfer process [86]. The idarubicin–dsDNA interaction was studied in incubated solutions and at multilayer dsDNA, poly(A), and poly(G) electrochemical biosensors [86]. The idarubicin intercalation into the dsDNA double helix was demonstrated via the decrease in G_r_ and A_r_ oxidation peak currents when increasing the incubation time, but no oxidative DNA damage related to the appearance of the 8-oxoG and 2,8-DHA oxidation peaks was observed.

Daunorubicin is an anthracycline antineoplastic antibiotic with therapeutic effects similar to those of doxorubicin. Its mechanism of action involves topoisomerase-mediated interaction with DNA, inhibiting DNA replication and repair and RNA and protein synthesis. A DNA electrochemical biosensor for daunorubicin was developed, based on a PGE modified with levan, a fructan homopolysaccharide biopolymer [87]. Daunorubicin detection in the linear range 10–40 μg mL^−1^, with LOD = 2.74 μg mL^−1^, was achieved. 

Epirubicin is an anthracycline antitumor antibiotic used to treat lymphomas and breast, ovarian, lung, and gastric cancers. A label-free DNA-based biosensor for epirubicin determination in biological samples, based on a PGE modified with polypyrrole, nitrogen-doped reduced graphene, and salmon sperm dsDNA, was developed [88]. Epirubicin was determined in the concentration range 0.004–55.0 μM, with LOD = 1.0 nM. The epirubicin intercalation into the dsDNA minor grooves and interaction with the G bases were confirmed by theoretical docking studies. 

Kanamycin is an antibiotic used to treat severe bacterial infections and tuberculosis. A DNA electrochemical biosensor for the detection of kanamycin, based on the absorption of 15-mer poly(C) sequences modified with graphene oxide onto a multiwalled CN-modified GCE, and further incubated in bovine serum albumin solutions, was developed [89]. The DPV detection of kanamycin, in the linear range 0.05 pM–100 nM, with LOD = 0.0476 pM, was achieved, with the DNA biosensor also showing good potential for the detection of antibiotic residues in food samples.

### 3.6. Antimetabolites

Antimetabolites are a large group of anticancer agents that structurally resemble substrates naturally produced by the body, but are different enough to interfere with their metabolism. The antimetabolites disrupt nucleic acid synthesis, and are thus very effective in the inhibition of the enzymatic processes of malignant cells. 

**Figure 6:**
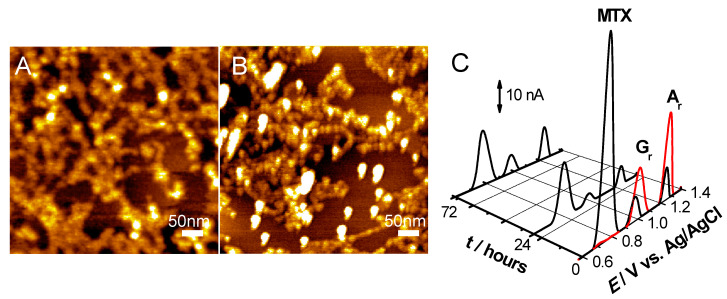
(**A**,**B**) AFM images at HOPG of (**A**) 10 μg mL^–1^ dsDNA and (**B**) 10 μg mL^–1^ dsDNA incubated with 0.5 μM methotrexate (MTX), for 24 h. (**C**) Baseline-corrected DPV at a GCE, at pH 4.5, of (red line) 100 µg mL^–1^ dsDNA and (black line) 100 µg mL^–1^ dsDNA incubated with 5 µM MTX, during different time periods. Adapted from [90] with permission.

Methotrexate (MTX) is an antimetabolite of folic acid that binds and inhibits the enzyme dehydrofolate reductase, and is used to treat psoriasis, rheumatoid arthritis, acute leukemia, head cancer, neck cancer, and osteosarcoma. The MTX–dsDNA interaction was studied by DPV at a GCE, and by AFM with HOPG, as shown in Figure 6 [90], showing that MTX induces dsDNA conformational modifications in a time-dependent manner. The decrease in the G_r_ and A_r_ residue oxidation peaks observed after short MTX–dsDNA incubation times was correlated with DNA double helix condensation and MTX intercalation. Using a dsDNA electrochemical biosensor, it was demonstrated that MTX induces dsDNA twisting and bending, followed by methotrexate intercalation and double helix unwinding. Using single-stranded poly(A) and poly(G) electrochemical biosensors, MTX’s preferential interaction with the A residues was established [90].

Raltitrexede is a folate analog antimetabolite, used to treat solid cancer tumors, such as malignant mesothelioma and gastric, head, neck, and pancreatic cancers, and is the main course of treatment for advanced colorectal cancer. In acid and physiological media, raltitrexede oxidation at a GCE takes place in two steps: the first step is pH independent and related to a one-electron transfer from the nitro group at the N10 position, by releasing a methyl cation, and the second step is pH dependent and related to a one-electron and one-proton transfer from the carbon at the C9 position, followed by a direct attack by a water molecule, leading to an irreversible dissociation of the oxidation product [91]. The raltitrexede–dsDNA interaction was studied with a DNA electrochemical biosensor, demonstrating raltitrexede intercalation into the double helix [91].

### 3.7. Kinase Inhibitors

The dysregulation of protein kinases, enzymes responsible for the process of phosphorylation, is involved in various processes of carcinogenesis, and therefore the development protein kinase inhibitors has been recognized to be useful in tumor therapy. 

Imatinib is an antineoplastic agent that inhibits the Bcr-Abl fusion protein tyrosine kinase, which is used to treat chronic myelogenous leukemia and gastrointestinal stromal tumors [92,93]. The dsDNA structural modifications after interaction with imatinib were studied in incubated solutions and with a dsDNA electrochemical biosensor [94], by monitoring the modifications of the G_r_ and A_r_ oxidation peak currents. Using poly(A) and poly(G) electrochemical biosensors, imatinib’s preferential binding to the A-rich segments was demonstrated, and an interaction mechanism was proposed. The electrochemically generated imatinib oxidation product within the dsDNA layer induced A residue oxidation, and the detection of the 2,8-DHA, biomarker of oxidative DNA damage was achieved [94].

**Figure 7:**
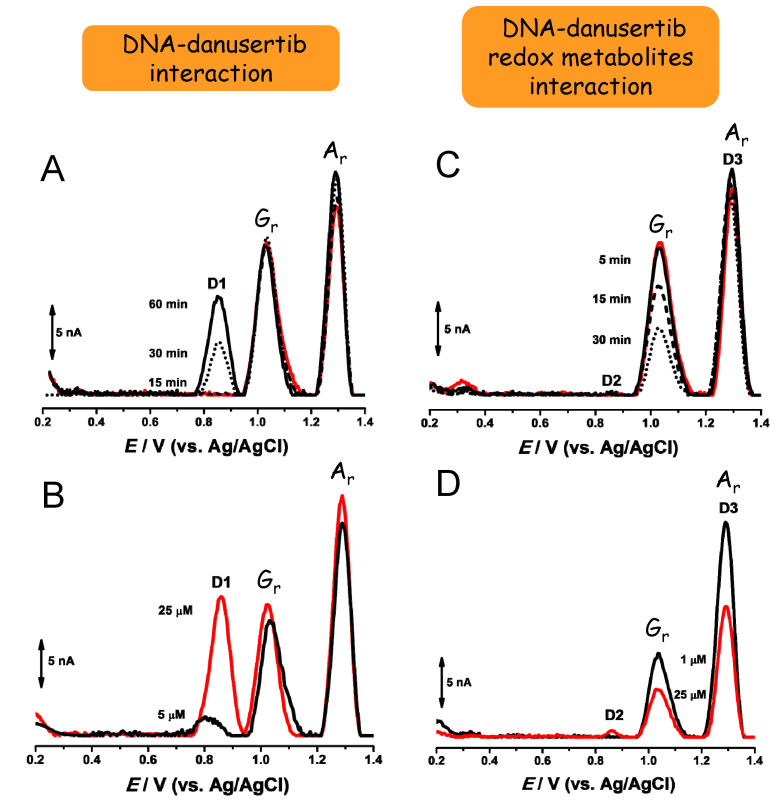
Baseline-corrected DPV at pH = 4.5, with a dsDNA electrochemical biosensor, (**A**,**B**) without conditioning potential and (**C**,**D**) with conditioning potential of +0.85 V (vs. Ag/AgCl, 3 M KCl): (**A**,**C**) (red line) 30 min in buffer, and (black lines) incubation in 10 µM danusertib for different time periods, and (**B**,**D**) incubation in (black line) 5 µM and (red line) 25 µM danusertib for 30 min. Adapted from [95] with permission.

Danusertib is an antineoplastic agent that inhibits the Aurora kinases, a family of serine–threonine kinases, and other tyrosine kinases, which are used for the treatment of various forms of tumors. The danusertib–dsDNA interaction was studied in incubated solutions and with a dsDNA electrochemical biosensor, as shown in Figure 7A,B [95]. Two sequential steps were observed: first, the danusertib positively charged piperazine moiety interacts electrostatically with the dsDNA negatively charged phosphate backbone, then, a danusertib–DNA complex that contains the danusertib pyrrolo-pyrazole moiety is formed, which causes small modifications of the DNA double helix, sensed by DPV via changes of G_r_ and A_r_ oxidation peaks [95].

The dsDNA interaction with an in situ electrochemically generated nitrenium cation radical was investigated, by controlling the danusertib oxidation at dsDNA, poly(G), and poly(A) electrochemical biosensors, as shown in Figure 7C,D [95]. A decrease in the G_r_ oxidation peak current was observed, which was correlated with the danusertib nitrenium cation radical redox metabolite binding at the C8 position in the G residues. Moreover, it was demonstrated that the danusertib–dsDNA interaction occurs mainly at the G-rich segments. Based on the formation danusertib redox metabolite–G adducts, the danusertib–dsDNA interaction mechanism was proposed [95]. 

Lapatinib is an inhibitor of the human epidermal growth factor receptor type 2 and epidermal growth factor receptor tyrosine kinases, and is used to treat breast cancer and other solid tumors. Lapatinib presents three oxidation peaks at a GCE, each consistent with a two electron transfer. The first oxidation peak is due to the (2-methylsulfonylethylamino)methyl oxidation to alcohol, and the second one is due to the 4-methoxyaniline oxidation to quinone imine [96]. The lapatinib–dsDNA interaction was studied using a DNA electrochemical biosensor, showing that lapatinib intercalates into the DNA double helix [96].

### 3.8. Immunomodulatory Agents

Thalidomide presents immunomodulatory, anti-inflammatory, antineoplastic, and antiangiogenic properties, and is currently used to treat erythema nodosum leprosum and various forms of cancer, mainly multiple myeloma. Voltammetric, AFM, UV–Vis, and electrophoresis studies showed that thalidomide intercalates into dsDNA, inducing time-dependent dsDNA structural modifications [97]. The thalidomide–dsDNA interaction was studied in incubated solutions and with a DNA electrochemical biosensor, by monitoring the thalidomide, G_r_, and A_r_ oxidation peaks, as a function of incubation time and concentrations of dsDNA and thalidomide. DPV showed that thalidomide presents affinity for both G and A residues. Initially, thalidomide induces DNA condensation, followed by thalidomide intercalation into the DNA, and double helix unwinding. The oxidative DNA damage caused by thalidomide was followed by monitoring the 8-oxoG and/or 2,8-DHA oxidation peaks.

Monoclonal antibodies (mAbs) are proteins that act like human antibodies in the immune system. Cancer treatment based on monoclonal antibodies is one of the most successful immunotherapeutic strategies, with mAbs presenting less severe side effects when compared with traditional chemotherapeutic drugs.

**Figure 8:**
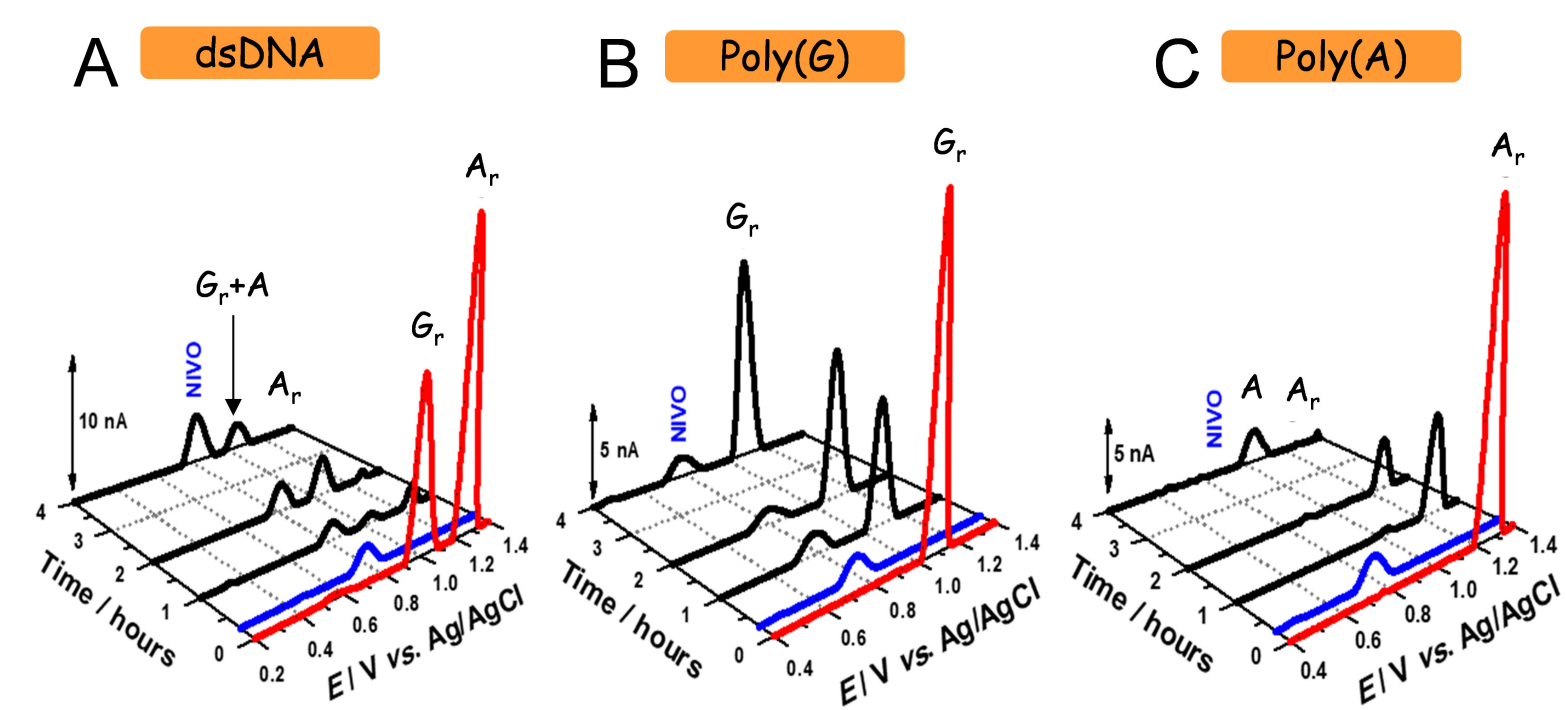
Baseline-corrected DPV at pH 4.5, for (blue line) control nivolumab (NIVO), (red line) control (**A**) dsDNA, (**B**) poly(G), and (**C**) poly(A) electrochemical biosensors and (black line) after incubation with NIVO for different time periods. Adapted from [98] with permission.

Nivolumab (NIVO) is an mAb used for the immunotreatment of metastatic melanoma, non-small cell lung cancer, renal cell carcinoma, and Hodgkin lymphoma. The NIVO–dsDNA interaction was investigated in incubated solutions, by DPV, UV–Vis spectrophotometry, gel electrophoresis, and in situ with dsDNA, poly(G), or poly(A) electrochemical biosensors, as shown in Figure 8 [98]. The modifications caused by NIVO in the electrochemical biosensor morphological structure were confirmed by DPV, EIS, and quartz crystal microbalance. The NIVO binding to dsDNA results in the formation of NIVO–dsDNA complexes, followed by the relaxation/unwinding of the dsDNA structure and the occurrence of abasic sites after the release of A bases from the dsDNA structure. However, the results showed that NIVO did not induce oxidative DNA damage.

Rituximab is a human/murine chimeric mAb that specifically binds to the transmembrane protein CD20 of B cells. DPV at a GCE demonstrated that native rituximab oxidation occurs at the tyrosine and tryptophan residues, while denatured rituximab oxidation occurs at tyrosine, tryptophan, and histidine residues [99]. The rituximab–dsDNA interaction was studied in incubated solutions and with a multilayer dsDNA electrochemical biosensor [100], demonstrating that rituximab induces the condensation of the DNA double helix, identified by the decrease and disappearance of the A_r_ oxidation peak current, the G_r_ oxidation peak current decrease, and the appearance of free G and A base oxidation peaks. However, rituximab did not cause oxidative DNA damage, as 8-oxoG and 2,8-DHA oxidation peaks did not occurred.

**Figure 9:**
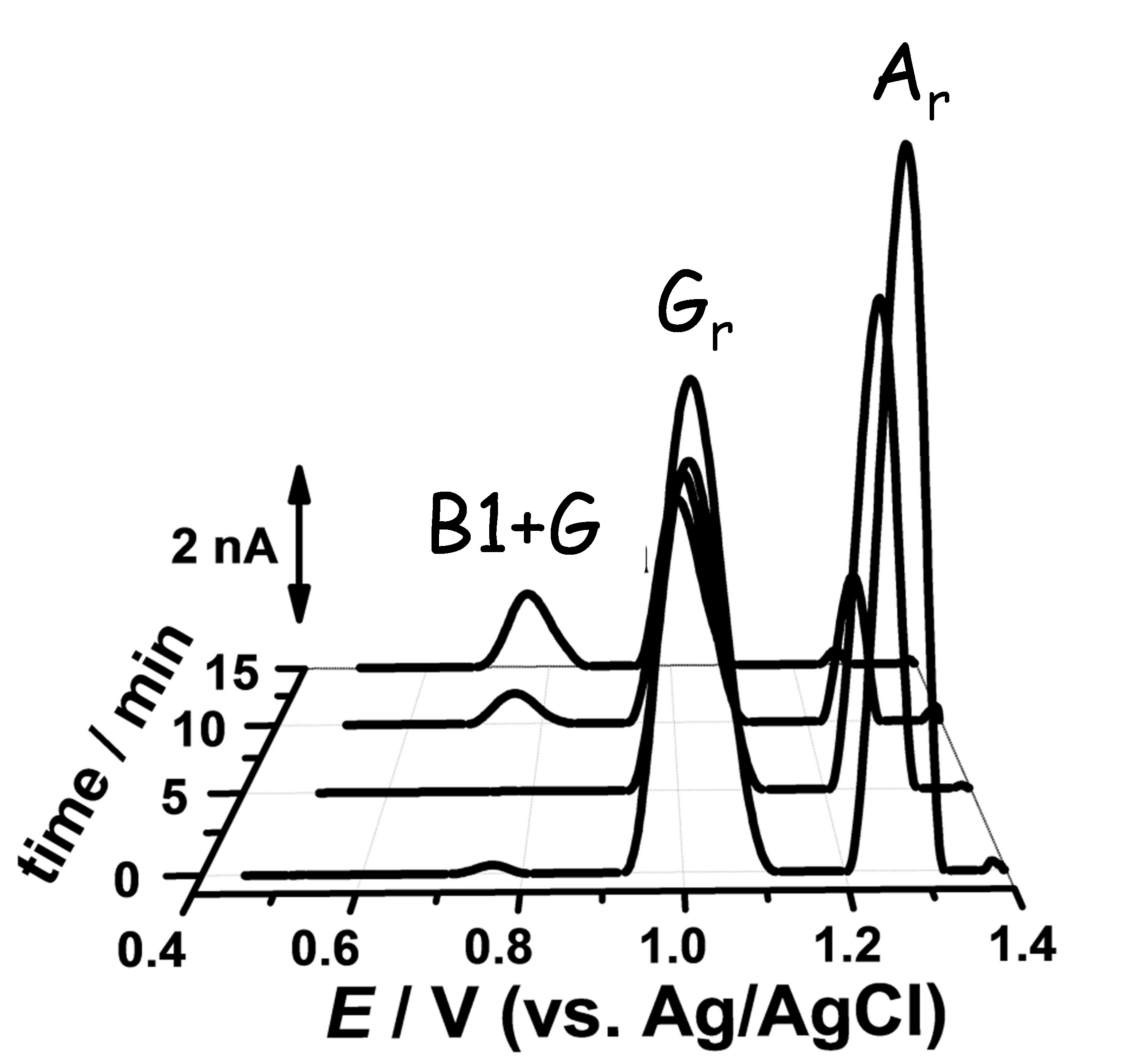
Baseline-corrected DPV at pH 4.5, with a dsDNA electrochemical biosensor incubated in 500 μg mL^−1^ bevacizumab for different time periods. Adapted from [102] with permission.

Bevacizumab, sold under the brand name Avastin, is an mAb used to treat colon, lung, kidney, cervical, breast, ovarian, and brain cancers. DPV studies with GCEs indicated that native bevacizumab presents one pH-dependent oxidation peak, due to tyrosine and tryptophan amino acid residue oxidation, while bevacizumab denatured by chemical agents presents several oxidation peaks, due to cysteine and histidine amino acid residue oxidation [101]. The bevacizumab–dsDNA interaction was studied in incubated solutions and with a dsDNA electrochemical biosensor [102]. A decrease and disappearance of the G_r_ and A_r_ oxidation peaks following the interaction were observed, due to adduct formation that hinders the purine bases, impeding their electron transfer, as shown in Figure 9. Increasing the incubation time, the free G base oxidation peak occurred, but no oxidative DNA damage was observed [102].

### 3.9. Nucleoside Analogs

Nucleoside analogs are pharmaceutical drugs with cytotoxic, immunosuppressive, and antiviral properties that are used as chemotherapeutic agents, especially for the treatment of leukemia. They are structurally similar to natural nucleosides, thus, they are easily incorporated into DNA or RNA by cells. 

Clofarabine and cladribine are second-generation adenosine analogs with antineoplastic activity, and are used to treat different types of leukemia. Both clofarabine [103] and cladribine [104] present, at a GCE, irreversible and pH-dependent oxidation processes that follow diffusion-controlled mechanisms and occur with the transfer of two protons and two electrons. Clofarabine [103] and cladribine [104] in situ interactions with DNA were investigated with dsDNA, poly(G), and poly(A) electrochemical biosensors, showing that both adenosine analogs cause time-dependent modifications of the DNA double helix, but no oxidative DNA damage was observed. 

Fludarabine is a nucleoside analog used to treat hairy cell leukemia, chronic lymphocytic leukemia, and several other hematopoietic malignancies. Fludarabine presents a one-proton and one-electron transfer oxidation process at GCEs, resulting in the formation of hydroxylated species [105]. The fludarabine–DNA interaction was investigated in incubated solutions and with dsDNA, poly(G), and poly(A) electrochemical biosensors, showing that fludarabine can cause modifications of the DNA double helix.

The cytosine analog gemcitabine’s electrochemical behavior was investigated, showing no electroactivity with GCEs [106]. The gemcitabine–DNA interaction with a dsDNA electrochemical biosensor leads to DNA structural modifications and damage, detected via changes of the G_r_ and A_r_ oxidation peak currents, and the occurrence of the free G base oxidation peak. The gemcitabine–DNA interaction mechanism showed two consecutive steps, the first one being independent of the DNA sequence, leading to the condensation/aggregation of the dsDNA, and the second one resulting in the formation of a gemcitabine–DNA rigid complex, that favors the interaction of the fluorine atoms of the gemcitabine ribose moiety with the G residues, causing the liberation of free G bases [106].

### 3.10. Metal Complexes

Metal-based compounds are extensively used to treat of various conditions, and the discovery of cisplatin represented a breakthrough in the history of metal-based compounds used for cancer treatment. As substitutes for this first-generation cisplatin anticancer agent, more recently, polynuclear metal complexes were synthetized, representing a third-generation class of anticancer agents. In particular, the synthesis of Pd(II) complexes was intended to obtain less toxic compounds, with increased tumor selectivity and improved solubility, comparing with the Pt(II) complexes [107]. The dsDNA interaction with the polynuclear Pd(II) chelates with the biogenic polyamines spermidine (Spd) and spermine (Spm) has been investigated [108]. Following the interaction, a decrease in the G_r_ and A_r_ residue oxidation peaks currents was detected, demonstrating that a Pd(II)-DNA adduct, stabilized by electrostatic interactions between the Spd and Spm polycationic chains and the DNA negatively charged phosphate backbones, is formed, restructuring the dsDNA absorbed layer. The Pd(II)–Spd complex interacts strongly with the dsDNA, since it presents three Pd(II) ions and two Spd chains (six possible coordination sites), compared with the Pd(II)–Spm complex with two Pd(II) ions and one Spm chain (four possible coordination sites). The Pd(II) polyamine complex–dsDNA interactions caused no oxidative DNA damage [108].

The lipoic acid–palladium complex (LAPd) that consists of a palladium bonded to both end-groups of a lipoic acid, has been developed as a non-toxic chemotherapeutical drug, in a pharmaceutical compound known as DNA reductase. The dsDNA interaction with LAPd and with Poly-MVA^TM^, a nutritional food supplement that contains the LAPd polymer as a trimer linked to a thiamine, was investigated [109], showing that both LAPd and Poly-MVA^TM^ interact with dsDNA, without causing oxidative DNA damage.

### 3.11. Phenolic Compounds

Natural phenolic compounds are abundant in the vegetable kingdom, occurring mainly as secondary metabolites in a wide variety of chemical structures, and many of them present antibiotic, antiviral, antifungal, antiparasitic, anti-inflammatory, antiproliferative, and cytotoxic activities [110]. 

Biflorin is an *ortho*-naphthoquinone synthetized from the plant *Capraria biflora*, indigenous to the West Indies and South America, and presents cytotoxic, antimicrobial, antitumor, and antimutagenic proprieties, and is used to treat skin, breast, and colon cancer. Biflorin’s pharmacokinetics were investigated by electrochemistry and spectrophotometry, showing that biflorin’s interaction with dsDNA and ssDNA takes place through intercalation [111]. 

Lawsone is a 1,4-napthoquinone derivate that presents antibacterial, antifungal, antiviral, and antineoplastic activities. Its capacity to inhibit tumor cell growth is related to the formation of reactive oxygen species (ROS). Lawsone–copper(II) complexes present even higher cytotoxic activity and have also been evaluated as possible drugs in cancer research [112]. The redox behavior of seven ternary copper(II) complexes of lawsone with O-donor (water) and N-donor ligands (pyridine, 2-, 3-, and 4-aminopyridine, 3-hydroxypyridine, and 3,5- dimethylpyrazole) has been investigated by CV and DPV [112]. Their interaction with DNA was also investigated with a DNA electrochemical biosensor prepared with CPEs. All lawsone complexes interacted with dsDNA; the simplest complex was Cu(lawsone)_2_(H_2_O)_2_·0.5 H_2_O that also acted as a pro-oxidant, increasing its cytotoxicity. 

Quercetin is a phenolic compound that belongs to the flavonoid class and exhibits anticancer properties, inhibiting a broad range of cancers, such as breast, lung, nasopharyngeal, kidney, colorectal, prostate, pancreatic, and ovarian cancers. The quercetin–dsDNA interaction was investigated with two types of DNA electrochemical biosensors [113], showing that quercetin undergoes oxidation even after dsDNA binding. After quercetin intercalation, its oxidation leads to the formation of radicals that cause breaks in the dsDNA hydrogen bonds, causing dsDNA damage, which was electrochemically detected via the appearance of an 8-oxoG oxidation peak. A mechanism for the oxidized quercetin–dsDNA interaction was proposed. In solution, the quercetin–dsDNA interaction was very weak [114]. DPV studies showed that the DNA–Cu(II)–quercetin interaction in solution leads to dsDNA time-dependent damage, demonstrated by the increase in the G_r_ and A_r_ oxidation peak currents, and corroborated by spectrophotometry studies [114]. 

## 4. Conclusions

Understanding drug interaction mechanisms with genomic DNA, in order to predict their structure–function mechanism of action, and performing analytical determination of drugs in pharmaceutical formulations, in human biologic fluids, or in wastewater, are now important tasks in medical, pharmaceutical, and environmental sciences. Due to the increased need for practical and low-cost analytical techniques for drug determination, electrochemical biosensor devices have gain increasing attention. 

The DNA electrochemical biosensors present not only the advantage of high sensitivity, short time of analysis, low cost, and shorter and simpler sample preparation steps, but also allow the in situ real-time determination of the DNA interaction mechanisms with the drug’s active component in pharmaceutical formulations, with its degradation products, with its redox reaction products, or with its metabolites in biological fluids. 

This review presents the design of DNA electrochemical biosensors based on the direct detection of DNA electrochemistry, for screening the oxidative DNA damage caused by pharmaceutical drugs. The AFM and voltammetric characterizations of the bottom-up immobilization procedures of self-assembled DNA nanostructures at the surface of the electrodes were described. Moreover, the review focuses on the applications of these DNA electrochemical biosensors in drug discovery and analysis, which may lead to more efficient screening systems in the future. The detection of DNA interactions with a wide range of pharmaceutical compounds, such as acridine derivatives, alkaloids, alkylating agents, alkylphosphocholines, antibiotics, antimetabolites, kinase inhibitors, immunomodulatory agents, metal complexes, nucleoside analogs, and phenolic compounds, were reviewed.

## Data Availability

Not applicable.

## References

[1] Kino K., Hirao-Suzuki M., Morikawa M., Sakaga A., Miyazawa H. (2017). Generation, repair and replication of guanine oxidation products. Genes Environ..

[2] Goyal R.N., Jain N., Garg D.K. (1997). Electrochemical and enzymic oxidation of guanosine and 8-hydroxyguanosine and the effects of oxidation products in mice. Bioelectrochem. Bioenerg..

[3] Brett C.M.A., Oliveira-Brett A.M., Serrano S.H.P. (1994). On the adsorption and electrochemical oxidation of DNA at glassy carbon electrodes. J. Electroanal. Chem..

[4] Goyal R., Dryhurst G. (1982). Redox chemistry of guanine and 8-oxyguanine and a comparison of the peroxidase-catalyzed and electrochemical oxidation of 8-oxyguanine. J. Electroanal. Chem. Interfacial Electrochem..

[5] Yudkina A.V., Shilkin E.S., Endutkin A.V., Makarova A.V., Zharkov D.O. (2019). Reading and Misreading 8-oxoguanine, a Paradigmatic Ambiguous Nucleobase. Crystals.

[6] Loft S., Danielsen P., Løhr M., Jantzen K., Hemmingsen J.G., Roursgaard M., Karotki D.G., Møller P. (2012). Urinary excretion of 8-oxo-7,8-dihydroguanine as biomarker of oxidative damage to DNA. Arch. Biochem. Biophys..

[7] Labuda J., Oliveira-Brett A.M., Evtugyn G., Fojta M., Mascini M., Ozsoz M., Palchetti I., Paleček E., Wang J. (2010). Electrochemical nucleic acid-based biosensors: Concepts, terms, and methodology (IUPAC Technical Report). Pure Appl. Chem..

[8] Oliveira-Brett A.M., Serrano S.H.P., Piedade A.J.P., Compton R.G. (1999). Electrochemistry of DNA. Applications of Kinetic Modelling.

[9] Oliveira-Brett A.M., L. Gorton (2005). DNA-based biosensors. Biosensors and Modern Biospecific Analytical Techniques.

[10] Oliveira-Brett A.M., Chiorcea-Paquim A., Diculescu V.C., Piedade J. (2006). Electrochemistry of nanoscale DNA surface films on carbon. Med Eng. Phys..

[11] Chiorcea-Paquim A.-M., Oliveira S.C., Diculescu V.C., Oliveira-Brett A.M., Barcelo D., Hansen P.-D., Palchetti I. (2017). Applications of DNA-Electrochemical Biosensors in Cancer Research. Comprehensive Analytical Chemistry.

[12] Oliveira-Brett A.M., Grimes C.A., Dickey E.C., Pishko M.V. (2006). Electrochemistry for probing DNA damage. Encyclopedia of Sensors.

[13] Oliveira-Brett A.M., Diculescu V.C., Chiorcea-Paquim A.M., Serrano S.H.P. (2007). Electrochemical Sensor Analysis. Comprehensive Analytical Chemistry.

[14] Oliveira-Brett A.M., Bartlett P.N. (2008). Electrochemical DNA Assays. Bioelectrochemistry: Fundamentals, Experimental Techniques and Applications.

[15] Oliveira-Brett A.-M., Schmuki P., Virtanen S. (2009). Nanobioelectrochemistry. Electrochemistry at the Nanoscale, Nanostrutures Science and Technology.

[16] Oliveira-Brett A.M., Diculescu V.C., Chiorcea-Paquim A., Serrano S.H.P. (2007). Chapter 20 DNA-electrochemical biosensors for investigating DNA damage. Vibrational Spectroscopy for Plant Varieties and Cultivars Characterization.

[17] Diculescu V.C., Chiorcea-Paquim A., Oliveira-Brett A.M. (2005). Electrochemical DNA Sensors for Detection of DNA Damage. Sensors.

[18] Diculescu V.C., Chiorcea-Paquim A.-M., Oliveira-Brett A.M. (2016). Applications of a DNA-electrochemical biosensor. TrAC Trends Anal. Chem..

[19] Oliveira S.C.B., Diculescu V., Chiorcea-Paquim A.M., Oliveira-Brett A. (2018). Electrochemical Biosensors for DNA–Drug Interactions. Encyclopedia of Interfacial Chemistry.

[20] Lopes I.C., Oliveira-Brett A.M. (2017). Human Cytochrome P450 (CYP1A2)-dsDNA Interactionin situ Evaluation Using a dsDNA-electrochemical Biosensor. Electroanalysis.

[21] Diculescu V.C., Chiorcea-Paquim A., Eritja R., Oliveira-Brett A.M. (2011). Evaluation of the structure–activity relationship of thrombin with thrombin binding aptamers by voltammetry and atomic force microscopy. J. Electroanal. Chem..

[22] Chiorcea-Paquim A.-M., Santos P.V., Eritja R., Oliveira-Brett A.M. (2013). Self-assembled G-quadruplex nanostructures: AFM and voltammetric characterization. Phys. Chem. Chem. Phys..

[23] Oliveira S.C.B., Oliveira-Brett A.M. (2010). In situ evaluation of chromium–DNA damage using a DNA-electrochemical biosensor. Anal. Bioanal. Chem..

[24] Oliveira S.C.B., Corduneanu O., Oliveira-Brett A.M. (2008). In situ evaluation of heavy metal–DNA interactions using an electrochemical DNA biosensor. Bioelectrochemistry.

[25] Chiorcea-Paquim A., Corduneanu O., Oliveira S.C.B., Diculescu V.C., Oliveira-Brett A.M. (2009). Electrochemical and AFM evaluation of hazard compounds–DNA interaction. Electrochim. Acta.

[26] Lopes I.C., Santos P.V.F., Diculescu V.C., Peixoto F.M.P., Araújo M.C.U., Tanaka A.A., Oliveira-Brett A.M. (2012). Microcystin-LR and chemically degraded microcystin-LR electrochemical oxidation. Analyst.

[27] Santos P.V.F., Lopes I.C., Diculescu V.C., Oliveira-Brett A.M. (2012). DNA–Cyanobacterial Hepatotoxins Microcystin-LR and Nodularin Interaction: Electrochemical Evaluation. Electroanalysis.

[28] Enache T.A., Chiorcea-Paquim A.-M., Fatibello-Filho O., Oliveira-Brett A.M. (2009). Hydroxyl radicals electrochemically generated in situ on a boron-doped diamond electrode. Electrochem. Commun..

[29] Oliveira S.C.B., Oliveira-Brett A.M. (2010). Boron doped diamond electrode pre-treatments effect on the electrochemical oxidation of dsDNA, DNA bases, nucleotides, homopolynucleotides and biomarker 8-oxoguanine. J. Electroanal. Chem..

[30] Oliveira S.C.B., Oliveira-Brett A.M. (2012). In Situ DNA Oxidative Damage by Electrochemically Generated Hydroxyl Free Radicals on a Boron-Doped Diamond Electrode. Langmuir.

[31] Piedade J.A.P., Oliveira P.S.C., Lopes M.C., Oliveira-Brett A.M. (2006). Voltammetric determination of γ radiation-induced DNA damage. Anal. Biochem..

[32] Oliveira-Brett A.M., Piedade J.A.P., Silva L.A., Diculescu V. (2004). Voltammetric determination of all DNA nucleotides. Anal. Biochem..

[33] Oliveira S.C.B., Oliveira-Brett A.M. (2010). DNA-electrochemical biosensors: AFM surface characterisation and application to detection of in situ oxidative damage to DNA. Comb. Chem. High Throughput Screen..

[34] Chiorcea-Paquim A.M., Pontinha A.D.R., Oliveira-Brett A.M. (2014). Time-dependent polyguanylic acid structural modifications. Electrochem. Commun..

[35] Oliveira-Brett A.M., Piedade J.A.P., Serrano S.H.P. (2000). Electrochemical Oxidation of 8-Oxoguanine. Electroanalalysis.

[36] Oliveira-Brett A.M., Diculescu V., Piedade J. (2002). Electrochemical oxidation mechanism of guanine and adenine using a glassy carbon microelectrode. Bioelectrochemistry.

[37] David S.S., O’Shea V.L., Kundu S. (2007). Base-excision repair of oxidative DNA damage. Nat. Cell Biol..

[38] Venkateswarlu D., Leszczynski J. (1998). Tautomeric equilibria in 8-oxopurines: Implications for mutagenicity. J. Comput. Mol. Des..

[39] Drake D.M., Shapiro A.M., Wells P.G. (2019). Measurement of the Oxidative DNA Lesion 8-Oxoguanine (8-oxoG) by ELISA or by High-Performance Liquid Chromatography (HPLC) with Electrochemical Detection. Breast Cancer.

[40] Valavanidis A., Vlachogianni T., Fiotakis C. (2009). 8-hydroxy-2′ -deoxyguanosine (8-OHdG): A Critical Biomarker of Oxidative Stress and Carcinogenesis. J. Environ. Sci. Health Part C.

[41] Subramanian P., Dryhurst G. (1987). Electrochemical oxidation of guanosine formation of some novel guanine oligonucleosides. J. Electroanal. Chem. Interfacial Electrochem..

[42] Oliveira-Brett A.M., Matysik F.-M. (1997). Sonoelectrochemical studies of guanine and guanosine. Bioelectrochem. Bioenerg..

[43] Oliveira-Brett A.M., Da Silva L.A., Brett C.M.A. (2002). Adsorption of Guanine, Guanosine, and Adenine at Electrodes Studied by Differential Pulse Voltammetry and Electrochemical Impedance. Langmuir.

[44] Chiorcea A.-M., Oliveira-Brett A.M. (2002). Scanning probe microscopic imaging of guanine on a highly oriented pyrolytic graphite electrode. Bioelectrochemistry.

[45] Oliveira-Brett A.M., Brett C.M.A., Silva L. (2002). An impedance study of the adsorption of nucleic acid bases at glassy carbon electrodes. Bioelectrochemistry.

[46] Rebelo I., Piedade J.A.P., Oliveira-Brett A.M. (2004). Electrochemical determination of 8-oxoguanine in the presence of uric acid. Bioelectrochemistry.

[47] Gupta P., Oyama M., Goyal R.N. (2015). Electrochemical investigations of 8-hydroxydeoxyguanosine and its determination at an edge plane pyrolytic graphite electrode. RSC Adv..

[48] Langmaier J., Samec Z., Samcová E. (2003). Electrochemical Oxidation of 8-Oxo-2′-Deoxyguanosine on Glassy Carbon, Gold, Platinum and Tin(IV) Oxide Electrodes. Electroanalysis.

[49] Shibata J.H. (2001). Nucleic Acids: Structures, Properties, and Functions (Bloomfield, Victor A.; Crothers, Donald M.; Tinoco, Ignacio, Jr.; contributions from Hearst, John E.; Wemmer, David E.; Kollman, Peter A.; Turner, Douglas H.). J. Chem. Educ..

[50] Chiorcea-Paquim A., Santos P.V., Oliveira-Brett A.M. (2013). Atomic force microscopy and voltammetric characterisation of synthetic homo-oligodeoxynucleotides. Electrochim. Acta.

[51] Chiorcea-Paquim A.-M., Oliveira-Brett A.M. (2016). Guanine Quadruplex Electrochemical Aptasensors. Chemosensors.

[52] Chiorcea-Paquim A.-M., Oliveira-Brett A.M. (2014). Redox behaviour of G-quadruplexes. Electrochim. Acta.

[53] Chiorcea-Paquim A., Santos P., Diculescu V., Eritja R., Oliveira-Brett A. (2012). Electrochemical Characterization of Guanine Quadruplexes. Guanine Quartets.

[54] Diculescu V.C., Chiorcea-Paquim A., Eritja R., Oliveira-Brett A.M. (2010). Thrombin-Binding Aptamer Quadruplex Formation: AFM and Voltammetric Characterization. J. Nucleic Acids.

[55] Pontinha A.D.R., Chiorcea-Paquim A., Eritja R., Oliveira-Brett A.M. (2014). Quadruplex Nanostructures of d(TGGGGT): Influence of Sodium and Potassium Ions. Anal. Chem..

[56] Chiorcea A.-M., Oliveira-Brett A.M. (2004). Atomic force microscopy characterization of an electrochemical DNA-biosensor. Bioelectrochemistry.

[57] Oliveira-Brett A.M., Chiorcea A.-M. (2003). Effect of pH and applied potential on the adsorption of DNA on highly oriented pyrolytic graphite electrodes. Atomic force microscopy surface characterisation. Electrochem. Commun..

[58] Chiorcea-Paquim A., Piedade J.A.P., Wombacher R., Jäschke A., Oliveira-Brett A.M. (2006). Atomic Force Microscopy and Anodic Voltammetry Characterization of a 49-Mer Diels-Alderase Ribozyme. Anal. Chem..

[59] Oliveira-Brett A.M., Chiorcea A.-M. (2003). Atomic Force Microscopy of DNA Immobilized onto a Highly Oriented Pyrolytic Graphite Electrode Surface. Langmuir.

[60] Chiorcea-Paquim A., Diculescu V.C., Oretskaya T., Oliveira-Brett A.M. (2004). AFM and electroanalytical studies of synthetic oligonucleotide hybridization. Biosens. Bioelectron..

[61] Oliveira-Brett A.M., Chiorcea-Paquim A.-M. (2005). DNA imaged on a HOPG electrode surface by AFM with controlled potential. Bioelectrochemistry.

[62] Oliveira-Brett A.M., Chiorcea-Paquim A.M., Diculescu V.C., Oretskaya T. (2005). Synthetic oligonucleotides: AFM characterisation and electroanalytical studies. Bioelectrochemistry.

[63] Chiorcea-Paquim A., Oretskaya T.S., Oliveira-Brett A.M. (2006). Adsorption of synthetic homo- and hetero-oligodeoxynucleotides onto highly oriented pyrolytic graphite: Atomic force microscopy characterization. Biophys. Chem..

[64] Chiorcea-Paquim A., Oretskaya T.S., Oliveira-Brett A.M. (2006). Atomic force microscopy characterization of synthetic pyrimidinic oligodeoxynucleotides adsorbed onto an HOPG electrode under applied potential. Electrochim. Acta.

[65] Tadini-Buoninsegni F., Palchetti I. (2020). Label-Free Bioelectrochemical Methods for Evaluation of Anticancer Drug Effects at a Molecular Level. Sensors.

[66] Chiorcea-Paquim A., Pontinha A.D.R., Oliveira-Brett A.M. (2015). Quadruplex-targeting anticancer drug BRACO-19 voltammetric and AFM characterization. Electrochim. Acta.

[67] Pontinha A.D.R., Sparapani S., Neidle S., Oliveira-Brett A.M. (2013). Triazole–acridine conjugates: Redox mechanisms and in situ electrochemical evaluation of interaction with double-stranded DNA. Bioelectrochemistry.

[68] Chiorcea-Paquim A., Pontinha A.D.R., Eritja R., Lucarelli G., Sparapani S., Neidle S., Oliveira-Brett A.M. (2015). Atomic Force Microscopy and Voltammetric Investigation of Quadruplex Formation between a Triazole-Acridine Conjugate and Guanine-Containing Repeat DNA Sequences. Anal. Chem..

[69] Javar H.A., Garkani-Nejad Z., Noudeh G.D., Mahmoudi-Moghaddam H. (2020). Development of a new electrochemical DNA biosensor based on Eu3+-doped NiO for determination of amsacrine as an anti-cancer drug: Electrochemical, spectroscopic and docking studies. Anal. Chim. Acta.

[70] Untiveros K.L., Da Silva E.G., De Abreu F.C., Da Silva-Júnior E.F., De Araújo-Junior J.X., De Aquino T.M., Armas S.M., De Moura R.O., Mendonça-Junior F.J., Serafim V.L. (2019). An electrochemical biosensor based on Hairpin-DNA modified gold electrode for detection of DNA damage by a hybrid cancer drug intercalation. Biosens. Bioelectron..

[71] Mahmoudi-Moghaddam H., Tajik S., Beitollahi H. (2019). A new electrochemical DNA biosensor based on modified carbon paste electrode using graphene quantum dots and ionic liquid for determination of topotecan. Microchem. J..

[72] Javar H.A., Mahmoudi-Moghaddam H. (2020). A Label-Free DNA Biosensor for Determination of Topotecan as an Anticancer Drug: Electrochemical, Spectroscopic and Docking Studies. J. Electrochem. Soc..

[73] Bolat G. (2020). Investigation of poly(CTAB-MWCNTs) composite based electrochemical DNA biosensor and interaction study with anticancer drug Irinotecan. Microchem. J..

[74] Vajedi F.S., Dehghani H. (2020). A high-sensitive electrochemical DNA biosensor based on a novel ZnAl/layered double hydroxide modified cobalt ferrite-graphene oxide nanocomposite electrophoretically deposited onto FTO substrate for electroanalytical studies of etoposide. Talanta.

[75] Ghalkhani M., Fernandes I.P.G., Oliveira S.C.B., Shahrokhianab S., Oliveira-Brett A.M. (2010). Electrochemical Redox Behaviour of Temozolomide Using a Glassy Carbon Electrode. Electroanalysis.

[76] Lopes I.C., De Oliveira S.C.B., Oliveira-Brett A.M. (2013). Temozolomide chemical degradation to 5-aminoimidazole-4-carboxamide – Electrochemical study. J. Electroanal. Chem..

[77] Lopes I.C., Oliveira S.C.B., Oliveira-Brett A.M. (2012). In situ electrochemical evaluation of anticancer drug temozolomide and its metabolites–DNA interaction. Anal. Bioanal. Chem..

[78] Jahandari S., Taher M.A., Karimi-Maleh H., Khodadadi A., Faghih-Mirzaei E. (2019). A powerful DNA-based voltammetric biosensor modified with Au nanoparticles, for the determination of Temodal; an electrochemical and docking investigation. J. Electroanal. Chem..

[79] De Carvalho P.A.V., Lopes I.C., Silva E.H.C., Bruzaca E.E.S., Alves H.J., Lima M.I.S., Tanaka A.A., Chaves H.J. (2019). Electrochemical behaviour of anticancer drug lomustine and in situ evaluation of its interaction with DNA. J. Pharm. Biomed. Anal..

[80] Machini W.B.S., Oliveira-Brett A.M. (2018). Antileishmanial Drug Miltefosine-dsDNA Interaction in situ Evaluation with a DNA-Electrochemical Biosensor. Electroanalysis.

[81] Hortobágyi G.N. (1997). Anthrazykline in der Krebstherapie. Drugs.

[82] Piedade J., Fernandes I., Oliveira-Brett A.M. (2002). Electrochemical sensing of DNA–adriamycin interactions. Bioelectrochemistry.

[83] Oliveira-Brett A.M., Piedade J.A.P., Chiorcea A.-M. (2002). Anodic voltammetry and AFM imaging of picomoles of adriamycin adsorbed onto carbon surfaces. J. Electroanal. Chem..

[84] Oliveira-Brett A.M., Vivan M., Fernandes I.R., Piedade J.A.P. (2002). Electrochemical detection of in situ adriamycin oxidative damage to DNA. Talanta.

[85] Kulikova T., Porfir’Eva A.V., Evtugyn G., Hianik T. (2019). Electrochemical DNA Sensors with Layered Polyaniline—DNA Coating for Detection of Specific DNA Interactions. Sensors.

[86] Kara H.E. (2014). Şatana Redox mechanism of anticancer drug idarubicin and in-situ evaluation of interaction with DNA using an electrochemical biosensor. Bioelectrochemistry.

[87] Congur G., Eksin E., Erdem A. (2021). Levan modified DNA biosensor for voltammetric detection of daunorubicin-DNA interaction. Sensors Actuators B Chem..

[88] Khodadadi A., Faghih-Mirzaei E., Karimi-Maleh H., Abbaspourrad A., Agarwal S., Gupta V.K. (2019). A new epirubicin biosensor based on amplifying DNA interactions with polypyrrole and nitrogen-doped reduced graphene: Experimental and docking theoretical investigations. Sensors Actuators B Chem..

[89] He X., Han H., Shi W., Dong J., Lu X., Yang W., Lu X. (2020). A label-free electrochemical DNA biosensor for kanamycin detection based on diblock DNA with poly-cytosine as a high affinity anchor on graphene oxide. Anal. Methods.

[90] Pontinha A.D.R., Jorge S.M.A., Paquim A.-M.C., Diculescu V.C., Oliveira-Brett A.M. (2011). In situ evaluation of anticancer drug methotrexate–DNA interaction using a DNA-electrochemical biosensor and AFM characterization. Phys. Chem. Chem. Phys..

[91] Queiroz N.L., Nascimento M.L., Nascimento J.A.M., Nascimento V.B., Oliveira S.C.B. (2018). Electrochemistry Study of Antineoplastic Raltitrexed Oxidation Mechanism and its Interaction with DNA. Electroanalysis.

[92] Diculescu V.C., Vivan M., Oliveira-Brett A.M. (2006). Voltammetric Behavior of Antileukemia Drug Glivec. Part I–Electrochemical Study of Glivec. Electroanalysis.

[93] Diculescu V.C., Vivan M., Oliveira-Brett A.M. (2006). Voltammetric Behavior of Antileukemia Drug Glivec. Part II—Redox Processes of Glivec Electrochemical Metabolite. Electroanalysis.

[94] Diculescu V.C., Vivan M., Oliveira-Brett A.M. (2006). Voltammetric Behavior of Antileukemia Drug Glivec. Part III: In Situ DNA Oxidative Damage by the Glivec Electrochemical Metabolite. Electroanalysis.

[95] Diculescu V.C., Oliveira-Brett A.M. (2016). In situ electrochemical evaluation of dsDNA interaction with the anticancer drug danusertib nitrenium radical product using the DNA-electrochemical biosensor. Bioelectrochemistry.

[96] Topal B.D., Bozal-Palabiyik B., Ozkan S.A., Uslu B. (2014). Investigation of anticancer drug lapatinib and its interaction with dsDNA by electrochemical and spectroscopic techniques. Sensors Actuators B Chem..

[97] Oliveira S.C.B., Chiorcea-Paquim A., Ribeiro S., Melo A., Vivan M., Oliveira-Brett A.M. (2009). In situ electrochemical and AFM study of thalidomide–DNA interaction. Bioelectrochemistry.

[98] Machini W.B.S., Marques N.V., Oliveira-Brett A.M. (2019). In Situ Evaluation of Anticancer Monoclonal Antibody Nivolumab-DNA Interaction Using a DNA-Electrochemical Biosensor. ChemElectroChem.

[99] Oliveira S.C.B., Santarino I., Oliveira-Brett A.M. (2013). Direct Electrochemistry of Native and Denatured Anticancer Antibody Rituximab at a Glassy Carbon Electrode. Electroanalysis.

[100] Santarino I., Oliveira S.C.B., Oliveira-Brett A.M. (2014). In Situ Evaluation of the Anticancer Antibody Rituximab-dsDNA Interaction Using a DNA-Electrochemical Biosensor. Electroanalysis.

[101] Issaad F.Z., Tomé L.I., Marques N.V., Mouats C., Diculescu V.C., Oliveira-Brett A.M. (2016). Bevacizumab anticancer monoclonal antibody: Native and denatured redox behaviour. Electrochim. Acta.

[102] Tomé L.I., Marques N.V., Diculescu V.C., Oliveira-Brett A.M. (2015). In situ dsDNA-bevacizumab anticancer monoclonal antibody interaction electrochemical evaluation. Anal. Chim. Acta.

[103] Satana H.E., Pontinha A.D.R., Diculescu V.C., Oliveira-Brett A.M. (2012). Nucleoside analogue electrochemical behaviour and in situ evaluation of DNA–clofarabine interaction. Bioelectrochemistry.

[104] Pontinha A.D.R., Satana H.E., Diculescu V.C., Oliveira-Brett A.M. (2011). Anodic Oxidation of Cladribine and In Situ Evaluation of DNA-Cladribine Interaction. Electroanalysis.

[105] Satana H.E., Oliveira-Brett A.M. (2011). In SituEvaluation of Fludarabine-DNA Interaction Using a DNA-Electrochemical Biosensor. Int. J. Electrochem..

[106] Buoro R.M., Lopes I.C., Diculescu V.C., Serrano S.H.P., Lemos L., Brett C.M.A. (2014). In situ evaluation of gemcitabine–DNA interaction using a DNA-electrochemical biosensor. Bioelectrochemistry.

[107] Corduneanu O., Chiorcea-Paquim A., Fiuza S.M., Marques M.P.M., Oliveira-Brett A.M. (2010). Polynuclear palladium complexes with biogenic polyamines: AFM and voltammetric characterization. Bioelectrochemistry.

[108] Corduneanu O., Chiorcea-Paquim A.-M., Diculescu V., Fiuza S.M., Marques M.P.M., Oliveira-Brett A.M. (2010). DNA Interaction with Palladium Chelates of Biogenic Polyamines Using Atomic Force Microscopy and Voltammetric Characterization. Anal. Chem..

[109] Corduneanu O., Chiorcea-Paquim A., Garnett M., Oliveira-Brett A.M. (2009). Lipoic acid–palladium complex interaction with DNA, voltammetric and AFM characterization. Talanta.

[110] Chiorcea-Paquim A., Enache T.A., Gil E.D.S., Oliveira-Brett A.M. (2020). Natural phenolic antioxidants electrochemistry: Towards a new food science methodology. Compr. Rev. Food Sci. Food Saf..

[111] Vasconcellos M.C., Costa C.D.O., Terto E.G.D.S., De Moura M.A.F., De Vasconcelos C.C., De Abreu F.C., De Lemos T.L.G., Costa-Lotufo L.V., Montenegro R.C., Goulart M.O.F. (2016). Electrochemical, spectroscopic and pharmacological approaches toward the understanding of biflorin DNA damage effects. J. Electroanal. Chem..

[112] Babula P., Vanco J., Krejcova L., Hynek D., Sochor J., Adam V., Trnkova L., Hubalek J., Kizek R. (2012). Voltammetric characterization of Lawsone-Copper(II) ternary complexes and their interactions with dsDNA. Int. J. Electrochem. Sci..

[113] Oliveira-Brett A.M., Diculescu V.C. (2004). Electrochemical study of quercetin–DNA interactions. Bioelectrochemistry.

[114] Oliveira-Brett A.M., Diculescu V.C. (2004). Electrochemical study of quercetin–DNA interactions: Part I. Analysis in incubated solutions. Bioelectrochemistry.

